# Twenty-year outcomes after repeat doses of antenatal corticosteroids prior to 32 weeks’ gestation: Follow-up of a randomised clinical trial

**DOI:** 10.1371/journal.pmed.1004618

**Published:** 2025-05-28

**Authors:** Robyn W. May, Anthony G. B. Walters, Greg D. Gamble, Caroline A. Crowther, Stuart R. Dalziel, Carl L. Eagleton, Christopher J. D. McKinlay, Barry J. Milne, Jane E. Harding

**Affiliations:** 1 Liggins Institute, University of Auckland, Auckland, New Zealand; 2 Department of Paediatrics: Child and Youth Health, University of Auckland, Auckland, New Zealand; 3 Department of Surgery, University of Auckland, Auckland, New Zealand; 4 Children’s Emergency Department, Starship Children’s Hospital, Auckland, New Zealand; 5 Centre of Methods and Policy Application in Social Sciences, University of Auckland, Auckland, New Zealand; University of Leeds, UNITED KINGDOM OF GREAT BRITAIN AND NORTHERN IRELAND

## Abstract

**Background:**

For women who have received a course of antenatal corticosteroids ≥7 days prior and have ongoing risk of preterm birth within the next 7 days, repeat dose(s) of corticosteroids up to 32 weeks’ gestation have been shown to reduce neonatal respiratory distress syndrome and serious health problems in the neonatal period but not other neonatal morbidities such as chronic lung disease, death, severe intraventricular haemorrhage or necrotising enterocolitis. Repeat antenatal corticosteroids were not associated with either benefit or harms in mid-childhood. However, this may have been too early to evaluate potential adverse effects on respiratory and other long-term outcomes. We aimed to assess if exposure to repeat dose(s) of antenatal corticosteroids administered to pregnant women up to 32 weeks’ gestation has beneficial or harmful effects on respiratory and general health of the offspring in adulthood.

**Methods and findings:**

We assessed the adult offspring of New Zealand participants in the Australasian Collaborative Trial of Repeat Doses of Corticosteroids for the Prevention of Neonatal Respiratory Disease (ACTORDS), a multicentre, placebo-controlled trial where women at risk of preterm birth within the next week, 7 or more days after having received a single course of corticosteroids were randomised to a repeat dose of intramuscular betamethasone or placebo, that could be repeated weekly if at ongoing preterm birth risk. Follow-up at 20 years included a health questionnaire and consent to access administrative data sources. The primary outcome was any asthma diagnosis. Secondary outcomes included neurodevelopmental, cardiovascular, mental and general health, functional difficulties and social outcomes. Of 352 infants born to 290 maternal trial participants, we assessed 214 (61%; 96 (45%) female) at mean (standard deviation) age 20.5 (1.5) years. The rate of any asthma diagnosis was similar in both groups (58/107 (54%) repeat bethamethasone versus 50/107 (47%) placebo; risk ratio adjusted for gestational age at trial entry, multiplicity and birth centre 1.13, 95% confidence interval, 0.87, 1.46). Differences between the groups for the secondary outcomes were generally small and confidence intervals included the possibility of no difference between groups.

**Conclusions:**

In this follow-up of a randomised clinical trial, our data suggest neither major harm nor benefit for the offspring in early adulthood following exposure to repeat dose(s) of antenatal corticosteroids compared with a single course prior to 32 weeks’ gestation. Smaller effects cannot be excluded and follow-up of adult offspring from other trials of repeat antenatal corticosteroids is recommended.

**Trial Registration:**

International Standard Randomized Controlled Trial, number ISRCTN48656428.

## Author summary

**Why was this study done?:**

- Antenatal corticosteroids given to women at risk of preterm birth reduce the baby’s risk of difficulty breathing after birth.
- For women who don’t give birth preterm but remain at risk, weekly repeat dose(s) of corticosteroids up to 32 weeks of pregnancy have also been shown to reduce the baby’s risk of difficulty breathing or having serious complications after birth and are not associated with any harms up to 8 years of age.
- However, there is concern that follow-up to 8 years may not have been long enough to evaluate potential harmful consequences for breathing and other health conditions.
- We aimed to assess if exposure to repeat dose(s) of antenatal corticosteroids given to pregnant women up to 32 weeks’ gestation has beneficial or harmful effects on lung conditions and general health of their children as adults.

**What did the researchers do and find?:**

- We assessed participants at 20 years of age who had been exposed to repeat dose(s) of antenatal corticosteroids or only one course followed by placebo during a randomised clinical trial.
- We used a health questionnaire and routinely collected administrative data to compare the rates of any asthma diagnosis, any other lung conditions, or any other general health conditions.
- We found no differences in any asthma diagnosis or any other conditions between those exposed to repeat antenatal corticosteroids and those who were not.

**What do these findings mean?:**

- Our data suggest neither benefit nor harm following exposure to repeat dose(s) of antenatal corticosteroids for the offspring 20 years later.
- The main limitations were the follow-up rate of only 61% and the use of health questionnaires and routinely collected data rather than in-person assessments.

## Introduction

For women at risk of preterm birth who have completed a course of corticosteroid treatment at least seven days previously and have ongoing risk of preterm birth prior to 32 weeks’ gestation, repeat doses of corticosteroids reduce neonatal respiratory distress syndrome and rates of serious health problems in the neonatal period [1,2]. In an individual participant data meta-analysis (IPD-MA) including 11 randomised trials, 4,857 women and 5,915 infants, the number needed to treat to prevent one infant from needing respiratory support was 21 (95% confidence interval 14–41) [2]. Available evidence could not exclude benefit or harm for other neonatal morbidities such as chronic lung disease, death, severe intraventricular haemorrhage or necrotising enterocolitis [1,2]. Repeat doses of antenatal corticosteroids are, therefore, recommended in several preterm birth management guidelines [3–7]. Although the evidence assessing long-term consequences of repeat antenatal corticosteroids is limited, follow-up until mid-childhood has not shown any long-term benefits or harms [1].

However, concerns remain about the potential harms of repeat antenatal corticosteroids, particularly since prediction of preterm birth is inaccurate and approximately 40% of those given antenatal corticosteroids go on to birth at term [8]. In this specific subgroup, a systematic review and meta-analysis of seven randomised controlled trials and 10 population based cohort studies (1.6 million infants total) found potential short- and long-term harms after term or late preterm births following antenatal corticosteroids exposure, including repeat doses [8]. The IPD-MA guidelines recommended limiting the number of repeat doses of antenatal corticosteroids to balance the beneficial respiratory effects with the dose-related reductions in weight, length, and head circumference *z*-scores at birth [2]. Recent reviews have highlighted that there is still much that is unknown about the potential benefits and harms of antenatal corticosteroids in different stages of pregnancy and different birth contexts [9] and that long-term consequences may be subtle and only manifest later in life [10]. Indeed, in the 2022 Cochrane review of repeat doses of antenatal corticosteroids, there were no trials with follow-up into adolescence or adulthood [1].

We therefore assessed the offspring of participants in the Australasian Collaborative Trial of Repeat Doses of Corticosteroids for the Prevention of Neonatal Respiratory Disease (ACTORDS) at 20 years of age to investigate the long-term effects of antenatal exposure to repeat doses of corticosteroids on respiratory and general health outcomes.

## Methods

### Ethics statement

Ethics approval was obtained from the Northern A Health and Disability Ethics Committee (20/NTA/37/AM02). Participants gave written informed consent.

### Study design

This was a follow-up of the adult offspring of New Zealand participants in the ACTORDS Trial (ISRCTN48656428), which is published [11], as are the results of the 2-year [12] and 6–8-year [13–16] follow-up assessments. The ACTORDS Trial was a multi-center, placebo-controlled, blinded, parallel group randomised clinical trial conducted from April 1998 to July 2004. Follow-up was conducted from January to October 2022.

Reporting of methods and results follow the Consolidated Standards of Reporting Trials (CONSORT) guideline [17] (S1 CONSORT Checklist).

### Participants

The ACTORDS Trial recruited women with a single, twin, or triple pregnancy at less than 32 weeks’ gestational age if they had received an initial course of corticosteroid seven or more days previously, and their responsible clinician considered them to be at continued risk of preterm birth within the next 7 days with no contraindication to further corticosteroid therapy. Women were excluded if they were in the second stage of labor, had chorioamnionitis needing urgent delivery, had mature lung development, or if further glucocorticoid therapy was judged to be essential. In this follow-up study, surviving offspring of women randomised in New Zealand who had not withdrawn from the original trial or follow-up studies were invited to complete a health questionnaire and consent to data linkage.

### Procedures

Participants were asked to complete a questionnaire online or in hard copy based on the New Zealand Health Survey 2018/2019 [18], which includes the Washington Group Short Set (WG-SS) questions on functional difficulties and activity limitations [19]. WG-SS responses were classified as no disability (“no difficulty” response to WG-SS questions), moderate disabilities (“some difficulty”) or severe disabilities (“a lot of difficulty” or “unable to do”) [20]. Consent was also sought to access administrative datasets managed by the New Zealand Ministry of Health [National Minimum Dataset (NMDS; hospital admissions and emergency department short stays (three hours or more) since 1988), Mortality dataset (MORT; national mortality records since 1970), Pharmaceutical dataset (PHARM; national records of community dispensing of pharmaceuticals funded by the New Zealand government since 1992)]; TestSafe (laboratory tests data for Auckland and Northland regions which include 36% of New Zealand’s population and New Zealand’s largest city where 61% of participants were born, since 2006); the Accident Compensation Corporation (ACC; national publicly funded personal injury insurance claims); Ministry of Education (MoE; national records of disciplinary measures during primary or secondary schooling); New Zealand Qualifications Authority (NZQA; national records of qualifications secondary school level and higher, since 1991); Ministry of Justice (MoJ, national records of convictions); and Whaikaha (Ministry of Disabled People; national records of support provided for persons with disabilities).

### Randomization and blinding

In the ACTORDS Trial, a central telephone randomisation service was used to assign women to a single intramuscular injection of either bethamethasone or a saline placebo packaged in identical, opaque study-labelled syringes. The study randomisation numbers were computer generated with variable block sizes, and stratification by center, gestational age (<28 weeks and ≥28 weeks), and number of fetuses (singleton, twin, or triplet). Participants, clinicians, and investigators were blinded to treatment allocation.

### Intervention

In the ACTORDS Trial, participants were randomised to intramuscular Celestone Chronodose, containing 7.8 mg betamethasone sodium phosphate and 6 mg betamethasone acetate (Schering-Plough, Sydney, Australia) or saline placebo, that could be repeated weekly until 32 weeks’ gestation if their responsible clinician considered the participant at continued risk of preterm birth within the next 7 days.

### Outcomes and measures

The primary outcome was any asthma diagnosis. Secondary outcomes included respiratory, neurodevelopmental, cardiovascular, diabetes and mental health composite outcomes, general and oral health, and disability status. Tertiary outcomes included educational and social outcomes and all components of the secondary composite outcomes. Outcome definitions and the data sources are summarized in Table 1 and the S1 Statistical Analysis Plan.

**Table 1:** Outcome definitions and the data sources from which they were derived.

	Data sources									
Outcomes	Self-report questionnaire	NMDS	PHARM	TestSafe	ACC	MoE	NZQA	MoJ	Whaikaha	MORT
**Primary outcome**										
Any asthma	x	x	x							
**Secondary outcomes**										
Asthma currently on treatment	x	x	x							
Death (any cause after randomization)										x
Respiratory composite	x	x								
Neurodevelopmental composite	x	x	x						x	
Cardiovascular composite	x	x	x							
Cardiovascular disease risk factors	x	x	x	x						
Diabetes composite	x	x	x	x						
Mental health composite	x	x	x		x					
Any bone disease	x	x								
Number of fractures	x	x			x					
Fair/poor general health	x									
Functional difficulties	x									
Fair/poor oral health	x									
**Tertiary outcomes**										
No secondary school qualification	x						x			
Any disciplinary action						x				
Any convictions								x		
Unemployment	x									
Alcohol use in the past year	x									
Recreational drug use	x									
Past/current smoking	x									
Past/current smoking pack years	x									
Past/current vaping	x									
*Components of respiratory composite outcome*										
Respiratory infections		x								
Chronic respiratory conditions		x								
Respiratory trauma		x								
Respiratory failure		x								
*Components of neurodevelopmental disability composite outcome*										
Visual impairment	x									
Hearing impairment	x	x								
Intellectual impairment	x	x							x	
Cerebral palsy	x	x							x	
Epilepsy	x	x	x							
Autism spectrum disorder	x	x							x	
Attention deficit hyperactivity disorder	x	x	x							
*Components of cardiovascular composite outcome*										
Hypertension	x	x	x							
Cardiomyopathies		x								
Arrhythmias		x								
Heart failure	x	x								
*Components of cardiovascular disease risk factors composite outcome*										
Dyslipidemia	x		x	x						
Diabetes mellitus composite	x	x	x	x						
Overweight/obesity	x	x								
*Components of diabetes composite outcome*										
Prediabetes	x			x						
Diabetes mellitus	x	x	x							
Gestational diabetes mellitus	x	x	x							
*Components of mental health composite outcome*										
Depression	x									
Bipolar affective disorder	x									
Anxiety disorders	x									
Suicide/self-harm					x					
*Components of functional difficulties outcomes*										
Difficulty seeing, even if wearing glasses	x									
Difficulty hearing, even if using a hearing aid	x									
Difficulty walking or climbing steps	x									
Difficulty remembering or concentrating	x									
Difficulty washing all over or dressing	x									
Difficulty communicating	x									
*Components of obesity/overweight outcome*										
Body mass index	x									
Height	x									
Weight	x									

ACC, Accident Compensation Corporation; MoE, Ministry of Education; MoJ, Ministry of Justice; MORT, Ministry of Health Mortality dataset; NMDS, Ministry of Health National Minimum Dataset; NZQA, New Zealand Qualifications Authority; PHARM, Ministry of Health Pharmaceutical dataset.

### Statistical analysis

The sample size was limited by the number of participants randomised in New Zealand and able to be located. For the primary outcome, the sample size of 214 allows for detection of a 22% increase in the proportion with any asthma diagnosis (relative risk 1.63) with 90% power (*β* = 0.10), assuming a baseline prevalence of 35% in the placebo group (based on the six to eight year follow-up for the whole trial cohort) [13].

All analyses were undertaken on an intention-to-treat basis according to a prespecified statistical analysis plan (S1 Statistical Analysis Plan) using R [21]. Denominators are number of participants with data available for the outcome, unless otherwise specified. There was no imputation for missing data, and participants for whom data were missing from all data sources for a specific outcome were excluded from that analysis.

Outcomes of participants exposed to repeat antenatal betamethasone or placebo were compared using generalised linear models. Continuous, binary, and count outcomes were analysed using Gaussian (identity link), binomial (log link) and Poisson (identity link) generalised linear models, respectively, and ordinal categorical variables were analysed using ordinal logistic regression. All models were adjusted for gestational age at trial entry, multiplicity and birth centre. Clustering due to multiple pregnancies was investigated using generalised linear mixed-effects regression models with inclusion of the unique maternal identifier as a random effect to account for the nonindependence of children from multiple pregnancies but produced similar estimates to the generalised linear models adjusted for multiplicity as a fixed effect (S1 Table). Prespecified subgroup analyses were performed for primary and secondary outcomes as main and interaction effects (with treatment allocation) for sex and socioeconomic status based on New Zealand Deprivation Index [22] for their area of residence at 20-year follow-up (most deprived (deciles 8–10) versus other). *P*-values < 0.05 for the interaction term were considered significant, but no adjustment for multiplicity was performed. Prespecified sensitivity analyses excluded participants with asthma diagnosed younger than 5 years old and no current asthma, and excluded participants with respiratory outcomes derived from hospital admission data where the respiratory diagnosis was not the primary reason for admission. During peer review, further sensitivity analyses on the primary outcome of any asthma diagnosis were requested including additional adjustment for maternal smoking at first antenatal visit and testing assumptions for the reasons for missing data for those who were not followed up. For the latter, pattern-mixture modeling within a multiple imputation framework was used [23] (S1 Text). A prespecified enriched sample analysis combined outcomes from the six to eight-year and the 20-year follow-up and the denominators were corrected to include the participants for whom data were available at either age.

## Results

### Cohort characteristics

**Fig 1:**
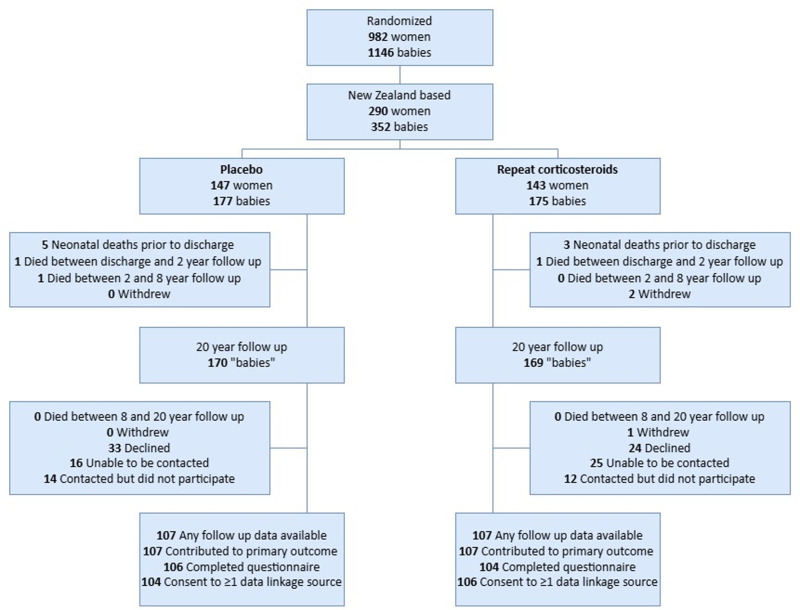
Participant flow diagram.

Of 352 infants born to 290 maternal trial participants recruited in New Zealand, 2 had withdrawn and 11 died (4 in the repeat bethamethasone group and 7 in the placebo group), leaving 339 eligible for 20-year follow-up. We assessed 214 (61%; 96 (45%) female) at mean (standard deviation) age 20.5 (1.5) years (Fig 1). Eligible offspring who did and did not participate had similar baseline characteristics, although the mothers of those not followed up were more likely to be multiparous (parity 4 + 19% versus 8.8%) and report smoking at their first antenatal visit (43% versus 26%; Table 2). There were also more Māori (30% versus 11%) or Pacific (13% versus 8.4%) and fewer European (50% versus 73%) mothers among those not followed up (Table 2). Those not followed up were more likely to have had a 5-min Apgar score <7 (8.0% versus 3.3%; Table 2). Baseline characteristics of the randomised groups in this follow-up cohort were similar, apart from reduced risk of neonatal respiratory syndrome, severe lung disease and need for respiratory support in the repeat betamethasone group, as reported previously[11] (Table 2).

**Table 2:** Characteristics of children, and their mothers, in the New Zealand ACTORDS Trial Cohort and 20-year follow-up.

Characteristic	Not followed up	Followed up		
		Total	Repeat	Placebo
Maternal characteristics at trial entry	*N* = 108	*N* = 182	*N* = 91	*N* = 91
Placebo	56 (52%)	91 (50%)		
Repeat	52 (48%)	91 (50%)		
Maternal age (years)	31 (27, 35)	32 (28, 35)	31 (27, 35)	32 (28, 34)
Parity				
0	33 (31%)	70 (38%)	38 (42%)	32 (35%)
1–3	55 (51%)	96 (53%)	49 (54%)	47 (52%)
4+	20 (19%)	16 (8.8%)	4 (4.4%)	12 (13%)
Multiple pregnancy	21 (19%)	38 (21%)	19 (21%)	19 (21%)
Smoking at first antenatal visit				
No	58 (54%)	130 (71%)	68 (75%)	62 (68%)
Unknown	4 (4%)	5 (3%)	3 (3%)	2 (2%)
Yes	46 (43%)	47 (26%)	20 (22%)	27 (30%)
Gestational age at entry (weeks)	28.6 (26.4, 30.3)	28.5 (26.1, 30.3)	28.4 (26.6, 30.6)	28.6 (25.9, 30.1)
Gestational age at first dose (weeks)	27.0 (25.3, 28.9)	26.6 (24.6, 28.7)	26.9 (24.9, 28.7)	26.1 (24.1, 28.6)
Main reasons for risk of preterm birth				
Antepartum haemorrhage	24 (22%)	42 (23%)	22 (24%)	20 (22%)
Cervical incompetence	4 (4%)	7 (4%)	4 (4%)	3 (3%)
Fetal growth restriction	8 (7%)	17 (9%)	8 (9%)	9 (10%)
Multiple pregnancies	2 (2%)	5 (3%)	3 (3%)	2 (2%)
Other	3 (3%)	8 (4%)	2 (2%)	6 (7%)
Preterm prelabour rupture of membranes	22 (20%)	42 (23%)	19 (21%)	23 (25%)
Pre/Eclampsia	25 (23%)	31 (17%)	15 (16%)	16 (18%)
Preterm labour	20 (19%)	30 (16%)	18 (20%)	12 (13%)
Caesarean section	69 (64%)	121 (66%)	63 (69%)	58 (64%)
**Infant characteristics at birth and neonatal outcomes**	***N* = 138**	***N* = 214**	***N* = 107**	***N* = 107**
Sex				
Female	69 (50%)	96 (45%)	43 (40%)	53 (50%)
Male	69 (50%)	118 (55%)	64 (60%)	54 (50%)
Maternal ethnicity				
Māori	41 (30%)	23 (11%)	11 (10%)	12 (11%)
Pacific	18 (13%)	18 (8%)	9 (8%)	9 (8%)
Asian	6 (4%)	10 (5%)	6 (6%)	4 (4%)
Other	4 (3%)	6 (3%)	2 (2%)	4 (4%)
European	69 (50%)	157 (73%)	79 (74%)	78 (73%)
Gestational age at birth (weeks)	31.0 (29.0, 34.0)	31.6 (29.6, 34.4)	32.0 (29.6, 35.0)	31.3 (29.6, 34.0)
Birth weight (g)	1,460 (1,118, 1,979)	1,653 (1,153, 2,198)	1,710 (1,117, 2,235)	1,600 (1,163, 2,195)
Birth weight *z*-scores	−0.50 (−1.25, 0.13)	−0.32 (−1.06, 0.34)	−0.43 (−1.15, 0.23)	−0.27 (−0.96, 0.39)
Respiratory distress syndrome	57 (41%)	81 (38%)	26 (24%)	55 (51%)
Severity of lung disease				
None	44 (32%)	68 (32%)	42 (39%)	26 (24%)
Mild	55 (40%)	83 (39%)	43 (40%)	40 (37%)
Moderate	21 (15%)	33 (15%)	15 (14%)	18 (17%)
Severe	18 (13%)	30 (14%)	7 (7%)	23 (21%)
Use of mechanical ventilation	38 (28%)	62 (29%)	23 (21%)	39 (36%)
Use of oxygen therapy	81 (59%)	120 (56%)	50 (47%)	70 (65%)
Use of surfactant	35 (25%)	59 (28%)	22 (21%)	37 (35%)
Serious neonatal morbidity	34 (25%)	44 (21%)	15 (14%)	29 (27%)
5-min Apgar score <7	11 (8%)	7 (3%)	3 (3%)	4 (3.7%)

Data are *n* (%) or median (IQR).

### Primary, secondary and tertiary outcomes

The risk of any asthma diagnosis was similar in both groups (58/107 (54%) repeat bethamethasone versus 50/107 (47%) placebo; adjusted risk ratio (aRR) 1.13, 95% confidence interval (CI), 0.87, 1.46, *p*-value = 0.35; Table 3).

**Table 3:** Primary and secondary outcomes.

Outcome	Repeat	Placebo	Unadjusted effect (95% CI)^a^	Adjusted effect (95% CI)^a^^,^^b^
**Primary outcome**				
Any asthma	58/107 (54%)	50/107 (47%)	1.16 (0.89, 1.51)	1.13 (0.87, 1.46)
**Secondary outcomes**				
Asthma currently on treatment	32/107 (30%)	33/107 (31%)	0.97 (0.65, 1.45)	0.97 (0.66, 1.43)
Death (any cause after randomisation)	4/175 (2.3%)	7/177 (4.0%)	0.58 (0.17, 1.94)	0.68 (0.2, 2.25)
Respiratory composite	40/105 (38%)	44/102 (43%)	0.88 (0.63, 1.23)	0.92 (0.68, 1.25)
Neurodevelopmental composite	24/107 (22%)	26/107 (24%)	0.92 (0.57, 1.50)	0.99 (0.62, 1.6)
Cardiovascular composite	12/107 (11%)	14/107 (13%)	0.86 (0.42, 1.77)	0.91 (0.44, 1.88)
Cardiovascular disease risk factors			0.94 (0.53, 1.64)	0.93 (0.52, 1.66)
0	71/107 (66%)	70/107 (65%)		
1	32/107 (30%)	31/107 (29%)		
> 1	4/107 (3.7%)	6/107 (5.6%)		
Diabetes composite	1/107 (0.9%)	3/107 (2.8%)	0.33 (0.04, 3.15)	0.31 (0.03, 2.86)
Mental health composite	34/107 (32%)	38/107 (36%)	0.89 (0.61, 1.3)	0.9 (0.62, 1.32)
Any bone disease	10/107 (9.3%)	8/107 (7.5%)	1.25 (0.51, 3.04)	1.17 (0.48, 2.83)
Number of fractures	0 (0, 7)	0 (0, 7)	−0.01 (−0.27, 0.25)	0.13 (−0.10, 0.36)
Fair/poor general health	13/104 (13%)	16/106 (15%)	0.83 (0.42, 1.63)	0.85 (0.43, 1.67)
Functional difficulties			0.81 (0.49, 1.35)	0.81 (0.49, 1.37)
No disability	39/104 (38%)	35/106 (33%)		
Moderate disability	47/104 (45%)	49/106 (46%)		
Severe disability	18/104 (17%)	22/106 (21%)		
Fair/poor oral health	19/104 (18%)	26/106 (25%)	0.74 (0.44, 1.26)	0.77 (0.45, 1.30)

Data are *n*/*N* (%) or median (minimum, maximum).

^a^Relative risk provided for binary outcomes, proportional odds ratios for categorical ordinal outcomes or mean difference for counts.

^b^Adjusted for gestational age at randomisation, multiplicity and birth centre.

CI, confidence interval.

Differences between the groups for the secondary outcomes were generally small and confidence intervals included the possibility of no difference between groups (Table 3). Of the 50% of the cohort who had ever been diagnosed with asthma, 30% were still being treated for asthma (Table 3). In both groups, there were similarly high rates of conditions contributing to the respiratory composite (40%), the mental health composite (34%), and functional difficulties classified as moderate (46%) and severe (19%; Table 3). Both groups had low rates of conditions contributing to the cardiovascular composite (12%) and the diabetes composite (1.9%) and two-thirds of participants had no cardiovascular risk factors (Table 3).

Differences between the repeat bethamethasone and placebo groups for the tertiary outcomes were generally small and confidence intervals included the possibility of no difference between groups (Table 4). Rates of alcohol and recreational drug use were similar between groups, as was past or current smoking (Table 4). Of conditions contributing to the neurodevelopmental disability composite, the repeat bethamethasone group had lower rates of visual (3.8% versus 5.7%; aRR 0.79, 95% CI, 0.23, 2.73), hearing (0% versus 3.7%) and intellectual impairment (3.8% versus 4.9%; aRR 0.95, 95% CI, 0.26, 3.38) and epilepsy (4.8% versus 5.9%; aRR 0.76, 95% CI, 0.23, 2.48) and higher rates of cerebral palsy (5.8% versus 2.0%; aRR 2.98, 95% CI, 0.60, 14.75) and autism spectrum disorder (6.5% versus 2.9%; aRR 2.56, 95% CI, 0.70, 9.38); None of these differences reached statistical significance. Although there were very few participants with diabetes outcomes, 2/3 (67%) of the offspring in the placebo group who had become pregnant had gestational diabetes compared to 0/8 of offspring in the repeat bethamethasone group. Weight, height, body mass index or rates of overweight and obesity were similar between groups and confidence intervals for the estimated mean difference included the possibility of no difference between groups (Table 4).

**Table 4:** Tertiary outcomes.

Outcome	Repeat	Placebo	Unadjusted effect (95% CI)^a^	Adjusted effect (95% CI)^a^^,^^b^
No secondary school qualification	14/107 (13%)	24/107 (22%)	0.58 (0.32, 1.07)	0.60 (0.33, 1.09)
Any disciplinary action	5/104 (4.8%)	12/101 (12%)	0.4 (0.15, 1.11)	0.41 (0.15, 1.13)
Any convictions	3/93 (3.2%)	1/85 (1.2%)	2.74 (0.29, 25.86)	2.33 (0.23, 23.51)
Unemployment	12/104 (12%)	19/106 (18%)	0.64 (0.33, 1.26)	0.6 (0.31, 1.18)
Alcohol use in the past year	82/104 (79%)	88/106 (83%)	0.95 (0.83, 1.08)	0.97 (0.86, 1.10)
Recreational drug use	34/104 (33%)	45/106 (42%)	0.77 (0.54, 1.10)	0.75 (0.53, 1.07)
Past/current smoking	21/104 (20%)	22/105 (21%)	0.96 (0.57, 1.64)	0.95 (0.56, 1.61)
Past/current smoking pack years	0.25 (0.00, 1.75)	1.00 (0.00, 6.00)	−1.48 (−2.23, −0.74)	−1.39 (−2.16, −0.62)
Past/current vaping	27/104 (26%)	37/106 (35%)	0.74 (0.49, 1.13)	0.73 (0.48, 1.10)
**Components of respiratory composite outcome**				
Respiratory infections	33/104 (32%)	35/102 (34%)	0.92 (0.63, 1.36)	0.96 (0.66, 1.37)
Chronic respiratory conditions	10/104 (9.6%)	10/102 (9.8%)	0.98 (0.43, 2.26)	1.1^*^
Respiratory trauma	1/104 (1.0%)	4/102 (3.9%)	0.25 (0.03,2.16)	0.23 (0.03, 2.03)
Respiratory failure	1/104 (1.0%)	2/102 (2.0%)	0.49 (0.05, 5.32)	0.96 (0.07, 13.55)
**Components of neurodevelopmental disability composite outcome**				
Visual impairment	4/104 (3.8%)	6/106 (5.7%)	0.68 (0.20,2.34)	0.79 (0.23,2.73)
Hearing impairment	0/107 (0%)	4/107 (3.7%)		
Intellectual impairment	4/105 (3.8%)	5/102 (4.9%)	0.78 (0.21, 2.81)	0.95 (0.26, 3.38)
Cerebral palsy	6/104 (5.8%)	2/102 (2.0%)	2.94 (0.61, 14.24)	2.98 (0.60, 14.75)
Epilepsy	5/104 (4.8%)	6/102 (5.9%)	0.82 (0.26,2.59)	0.76 (0.23, 2.48)
Autism spectrum disorder	7/107 (6.5%)	3/105 (2.9%)	2.29 (0.61, 8.62)	2.56 (0.70, 9.38)
Attention deficit hyperactivity disorder	11/107 (10%)	13/105 (12%)	0.83 (0.39, 1.77)	1.03 (0.50, 2.15)
**Components of cardiovascular composite outcome**				
Hypertension	9/107 (8.4%)	12/107 (11%)	0.75 (0.33, 1.71)	0.83 (0.37, 1.90)
Cardiomyopathies	1/104 (1.0%)	0/102 (0%)		
Arrhythmias	5/104 (4.8%)	3/102 (2.9%)	1.63 (0.40, 6.66)	1.69 (0.41, 7.00)
Heart failure	1/107 (0.9%)	0/107 (0%)		
**Components of cardiovascular disease risk factors composite outcome**				
Dyslipidaemia	6/107 (5.6%)	7/107 (6.5%)	0.86 (0.3, 2.47)	0.79 (0.28, 2.27)
Diabetes mellitus composite	1/107 (0.9%)	3/107 (2.8%)	0.33 (0.04, 3.15)	0.31 (0.03, 2.86)
Overweight/obesity	25/85 (29%)	25/74 (34%)	0.87 (0.55, 1.38)	0.96 (0.63, 1.47)
**Components of diabetes composite outcome**				
Prediabetes	1/103 (1.0%)	0/95 (0%)		
Diabetes mellitus	0/107 (0%)	1/107 (0.9%)		
Gestational diabetes mellitus	0/8 (0%)	2/3 (67%)		
**Components of mental health composite outcome**				
Depression	28/105 (27%)	22/106 (21%)	1.28 (0.79, 2.09)	1.32 (0.81, 2.14)
Bipolar affective disorder	1/106 (0.9%)	0/107 (0%)		
Anxiety disorders	28/106 (26%)	28/106 (26%)	1.00 (0.64, 1.57)	0.96 (0.61, 1.52)
Suicide/self-harm	1/102 (1.0%)	0/94 (0%)		
**Components of functional difficulties outcomes**				
Difficulty seeing, even if wearing glasses			1.10 (0.59, 2.08)	1.14 (0.60, 2.17)
No disability	78/104 (75%)	82/106 (77%)		
Moderate disability	22/104 (21%)	18/106 (17%)		
Severe disability	4/104 (3.8%)	6/106 (5.7%)		
Difficulty hearing, even if using a hearing aid			1.12 (0.42, 3.03)	1.11 (0.41, 3.05)
No disability	95/104 (91%)	98/106 (92%)		
Moderate disability	9/104 (8.7%)	5/106 (4.7%)		
Severe disability	0/104 (0%)	3/106 (2.8%)		
Difficulty walking or climbing steps			1.28 (0.63, 2.59)	1.36 (0.65, 2.82)
No disability	83/104 (80%)	89/106 (84%)		
Moderate disability	18/104 (17%)	12/106 (11%)		
Severe disability	3/104 (2.9%)	5/106 (4.7%)		
Difficulty remembering or concentrating			0.86 (0.51, 1.45)	0.84 (0.49, 1.44)
No disability	56/103 (54%)	53/106 (50%)		
Moderate disability	34/103 (33%)	39/106 (37%)		
Severe disability	13/103 (13%)	14/106 (13%)		
Difficulty washing all over or dressing			0.74 (0.23, 2.4)	0.68 (0.2, 2.33)
No disability	99/104 (95%)	99/106 (93%)		
Moderate disability	1/104 (1.0%)	5/106 (4.7%)		
Severe disability	4/104 (3.8%)	2/106 (1.9%)		
Difficulty communicating			1.04 (0.56, 1.92)	1.09 (0.58, 2.05)
No disability	77/104 (74%)	79/106 (75%)		
Moderate disability	21/104 (20%)	22/106 (21%)		
Severe disability	6/104 (5.8%)	5/106 (4.7%)		
**Components of obesity/overweight outcome**				
BMI	24.2 (7.7)	24.7 (7.3)	−0.44 (−2.79, 1.91)	−0.22 (−2.53, 2.08)
Height	174 (10)	171 (11)	2.70 (−0.48, 5.87)	3.03 (−0.19, 6.24)
Weight	73 (25)	73 (23)	0.22 (−7.04, 7.47)	0.93 (−6.13, 8)

Data are *n*/*N* (%) or mean (median; minimum, maximum).

^a^Relative risk provided for binary outcomes, proportional odds ratios for categorical ordinal outcomes or mean difference for counts or continuous outcomes.

^b^Adjusted for gestational age at randomisation, multiplicity and birth centre.

^c^In both groups, the past or current smoking pack years were low, with the exception of an outlier with a 6 pack-year smoking history in the placebo group.

*Confidence intervals for relative risk not calculable.

CI, confidence interval.

### Subgroup and exploratory analyses

The prespecified subgroup analysis for sex showed that males had increased rates of any asthma diagnosis in the repeat bethamethasone group (39/64 (61%) versus 23/54 (43%) placebo; aRR 1.43, 95% CI, 1.00, 2.03) but there were no group differences in females (19/43 (44%) versus 27/53 (51%); aRR 0.86, 95% CI, 0.56, 1.32; interaction *p*-value = 0.06 (S2 Table). However, there were no sex differences in the rates of asthma still requiring treatment at follow-up (males: 20/64 (31%) versus 16/54 (30%); aRR 1.03, 95% CI, 0.61, 1.76; females: 12/43 (28%) versus 17/53 (32%); aRR 0.91, 95% CI, 0.49, 1.67; interaction *p*-value = 0.74; S2 Table). The treatment effect and sex interactions for all of the other secondary outcomes included the possibility of no difference (S2 Table). Overall, there were higher rates of most outcomes in the most deprived subgroup compared to the less deprived subgroup but no evidence of treatment effect and deprivation interactions (S3 Table).

The prespecified sensitivity (S4 Table) and enriched sensitivity analyses (S5 Table) produced results similar to the initial analyses. In the post hoc analysis of sensitivity to missing data, multiple imputation was used to impute the primary outcome of any asthma diagnosis based on the available participant characteristics in Table 2 under the assumption of missingness at random where participants not lost to follow-up are exchangeable with those lost to follow-up. This produced similar results to the primary analysis (aRR 1.15, 95% CI 0.89, 1.48; *p*-value = 0.28). If participants who were not followed up were not missing at random and the reason for loss to follow-up was related to the primary outcome of asthma, the confidence intervals for all scenarios of increased or decreased risk of asthma in those not followed up still include the possibility of no difference between groups (S1 Fig). Although characteristics of participants in the repeat bethamethasone and placebo groups who were and were not followed up were similar, if for some reason those not followed up in the repeat bethamethasone group were at a reduced risk of asthma, this would still not change our conclusion (S1 Fig). If those not followed up in the repeat bethamethasone group were at more than 7% increased risk of asthma, the confidence interval would no longer contain the possibility of no difference between groups, suggesting that repeat bethamethasone is associated with an increased risk; however, we have no reason to believe this might be the case.

## Discussion

In this follow-up of a randomised clinical trial of repeat doses of antenatal corticosteroids given to women who remained at risk of very preterm birth at least seven days after an initial course of corticosteroids, our data suggest neither beneficial nor harmful effects on respiratory and general health outcomes in early adulthood in those exposed to repeat dose(s) of antenatal corticosteroids compared with placebo. This is the longest follow-up of a randomised controlled trial of repeat antenatal corticosteroids and suggests that the short-term benefits of repeat antenatal corticosteroids are not outweighed by long-term harms.

These reassuring findings are in keeping with previous systematic reviews of repeat antenatal corticosteroids, although these only included follow-up to five years [24] and six to eight years [1] respectively, which may have been too early to evaluate long-term adverse outcomes. In the five-year follow-up of the Multiple Courses of Antenatal Corticosteroids for Preterm Birth Study (MACS-5), no significant difference between the single and multiple courses groups in the risk of death or neurodevelopmental disability was found [25]. However, those exposed to repeat antenatal corticosteroids who were born at term had a 1.7-fold increased odds of death or disability at 5 years of age and an almost 4-fold increased odds of neurosensory disability [25]. The latter finding should be interpreted cautiously, as analyses based on post-randomisation variables can introduce bias and confounding, and gestational age at birth is a post-randomisation variable which cannot be predicted at the time of corticosteroid administration, and therefore cannot inform clinical practice. Observational studies have also suggested possible later adverse effects of antenatal corticosteroids. A non-randomised Australian cohort of 541 very preterm infants found reduced rates of cerebral palsy in those exposed to repeat antenatal corticosteroids, but increased rates of aggressive or destructive, distractible, and hyperkinetic behavior at both three and six years of age [26]. A Finnish population-based retrospective cohort study of 674,877 children at median 5.8 years found a higher risk of mental and behavioral disorders in those exposed to antenatal corticosteroids [27]. Although this study did not account for the number or timing of antenatal corticosteroids treatments, the cohort included children born between January 1, 2006, and December 31, 2017, spanning a Finnish guideline change in 2009 from not recommending repeat antenatal corticosteroids to recommending one repeat course when the risk of respiratory distress was high [27]. A Taiwanese population-based retrospective cohort study of 1,163,443 children followed up for at least six years also found a higher risk of mental disorders in those exposed to a single course of antenatal corticosteroids [28]. However, these observational studies should be interpreted with caution due to inevitable confounding by indication for corticosteroid treatment, particularly as follow-up of randomised controlled trials have not found any evidence for these adverse outcomes.

In this 20-year follow-up study, where all participants had been exposed to a single course of antenatal corticosteroids, the proportion of participants with neurodevelopmental disorders were similar between those who were and those who were not exposed to repeat betamethasone and confidence intervals included the possibility of no difference between groups, but rates of neurodevelopmental disorders and functional difficulties were high. Of all the domains in the Washington Group Short Set (WG-SS) questions, participants reported greatest difficulties in remembering or concentrating (27/209 (13%) reporting at least a lot of difficulty) and communication (11/210 (5%) reporting at least a lot of difficulty). The rate of participants reporting at least a lot of difficulty in at least one domain was high (40/210 (19%)), double the rates in a 2018 cross-sectional survey of randomly selected adults in the greater Christchurch region in New Zealand (259/2807 (9%)) [29]. This finding is in keeping with a small study which found an increased risk of impaired cognitive development in early childhood in those whose mothers had threatened preterm labor, regardless of whether they had received antenatal corticosteroids or went on to give birth at term [30]. Pregnancies which are compromised not only by an initial risk of preterm birth, but ongoing risk at least one week after administration of antenatal corticosteroids, may be a particularly high risk group for adverse neurodevelopmental outcomes later in life.

The long-term consequences of antenatal corticosteroid administration may be different in males and females. Preterm males are at higher risk than females of respiratory distress syndrome and bronchopulmonary dysplasia [31]. Although a 2010 systematic review and meta-analysis found no sex differences in neonatal outcomes following antenatal corticosteroids [32], more recent studies have found greater respiratory benefit for male neonates compared to females [33,34]. In the whole ACTORDS trial cohort, rates of any asthma diagnosis by six to eight years in males and females were similar (males: 182/477 (38%); females: 132/394 (34%)) but males were over-represented in the New Zealand cohort (males: 83/162 (51%); females: 53/147 (36%)). At 20-years in this cohort, we found rates of any asthma diagnosis were similar in males and females (males: 62/118 (53%); females: 46/96 (48%)), but males were over-represented in the repeat bethamethasone group (S2 Table). There was no association between treatment effect and sex for rates of asthma still requiring treatment at 20 years, suggesting that treatment effect and sex interactions, if present, tend to decrease over time. While the follow-up assessments of this cohort are underpowered to detect sex differences, the effect of sex on the long-term respiratory consequences of antenatal corticosteroid administration warrants further investigation.

One of the main longstanding concerns around antenatal corticosteroids, given that their mechanism of action is to shift cellular function from proliferation to maturation [35], is possible adverse effects on growth. Although those in the repeat bethamethasone group in the ACTORDS trial had lower *z*-scores for weight and head circumference at birth compared to placebo, by hospital discharge there were no differences [11]. Likewise, follow-up of the whole cohort at two years [12] and six to eight years [13] found no differences in body size measurements between groups, and body composition studies in the New Zealand cohort at six to eight years also found no differences [14]. Participants have attained full adult stature by 20-year follow-up and there is still no evidence of body size differences between those who were and were not exposed to repeat antenatal corticosteroids. These data provide reassurance that the observed effects of repeat antenatal corticosteroids on fetal growth are transient.

### Strengths and limitations

The strength of this follow-up study is that it is the longest follow-up of a randomised controlled trial of repeat antenatal corticosteroids. Any effects of repeat antenatal corticosteroids on asthma, mental health or growth are unlikely to change substantially beyond the age of 20 years. Limitations include the modest proportion of subjects followed up (61% of those eligible). While the study had sufficient power to detect a 22% increase in any asthma diagnosis in those exposed to repeat antenatal corticosteroids, which we would consider to be a clinically important difference, given the small sample size, we cannot exclude a smaller but potentially clinically important difference. Lower follow-up rates in those who may be at an increased risk of adverse outcomes (Māori and Pacific ethnicity, mothers smoking at their first antenatal visit and those with low Apgar scores at birth) could lead to underestimates of adverse outcomes and limit generalisability. However, our sensitivity to missing data analysis that accounted for these differences in those followed up or not provided similar results. Furthermore, there is no evidence that these characteristics were differential between randomised groups, and thus were unlikely to affect our conclusions. By restricting follow-up to the New Zealand cohort, we could increase validity of outcome ascertainment by using routinely collected individually identified health data. Although data linkage has limitations, we made use of both a self-reported health questionnaire and data linkage to derive outcomes, which increases detection of outcomes of interest compared to using either source alone [36] and does not lead to biased conclusions in the context of follow-up of a randomised controlled trial [37].

In conclusion, in this follow-up of a randomised clinical trial, our data suggest neither major harm nor benefit for the offspring in early adulthood following exposure to repeat dose(s) of antenatal corticosteroids compared with a single course prior to 32 weeks’ gestation. Smaller effects cannot be excluded and follow-up of adult offspring from other trials of repeat antenatal corticosteroids is recommended.

## Supporting information

S1 CONSORT Checklist ACTORDS 20-year follow-up CONSORT Checklist. (DOC)

S1 Table Comparison of outcomes using generalised linear models adjusted for multiplicity as a fixed effect and generalised linear mixed-effects regression models accounting for clustering due to multiple pregnancies. (DOCX)

S2 Table Subgroup analyses: sex. (DOCX)

S3 Table Subgroup analyses: deprivation. (DOCX)

S4 Table Sensitivity analyses. (DOCX)

S5 Table Enriched sample analysis. (DOCX)

S1 Fig Sensitivity to missing data analysis. (TIF)

S1 Text Supplementary methods for missing data sensitivity analysis. (DOCX)

S1 Statistical Analysis Plan The AnteNatal Corticosteroids Health Outcomes Review (ANCHOR) Study: Australasian Collaborative Trial of Repeat Doses of Steroids (ACTORDS) follow-up. (PDF)

## Abbreviations:

ACTORDS: Australasian Collaborative Trial of Repeat Doses of Steroids
aRR: adjusted risk ratio
CONSORT: Consolidated Standards of Reporting Trials
IPD-MA: individual participant data meta-analysis
MACS: Multiple Courses of Antenatal Corticosteroids for Preterm Birth Study
WG-SS: Washington Group Short Set
