# Supplementary material for: Twenty-year outcomes after repeat doses of antenatal corticosteroids prior to 32 weeks’ gestation: Follow-up of a randomised clinical trial
Source: PLoS Med. 2025 May 28;22(5):e1004618. doi: 10.1371/journal.pmed.1004618 (PMC12118977; doi:10.1371/journal.pmed.1004618)
Supplement: S1 Table — (DOCX) [file pmed.1004618.s002.docx]

S1 Table. Comparison of outcomes using generalized linear models adjusted for multiplicity as a fixed effect and generalized linear mixed-effects regression models accounting for clustering due to multiple pregnancies.

| **Outcome** | **Repeat** | **Placebo** | **GLM unadjusted effect (95% CI)^a^** | **GLM adjusted effect (95% CI)^a,b^** | **GLMM unadjusted effect (95% CI)^a,c^** | **GLMM adjusted effect (95% CI)^a,c,d^** |
| --- | --- | --- | --- | --- | --- | --- |
| **Primary outcome** | | | | | | |
| Any asthma | 50/107 (47%) | 58/107 (54%) | 1.16 (0.89,1.51) | 1.16 (0.89,1.51) | 1.18 (0.87,1.48) | 1.01 (0.99,1.02) |
| **Secondary outcomes** | | | | | | |
| Asthma currently on treatment | 32/107 (30%) | 33/107 (31%) | 0.97 (0.65,1.45) | 0.99 (0.66,1.47) | 0.97 (0.62,1.4) | 1 (0.97,1.02) |
| Death (any cause after randomisation) | 4/175 (2.3%) | 7/177 (4.0%) | 0.58 (0.17,1.94) | 0.65 (0.19,2.17) | 0.58 (0.17,1.94)* | 0.64 (0.18,2.26) |
| Respiratory composite | 40/105 (38%) | 44/102 (43%) | 0.88 (0.63,1.23) | 0.98 (0.72,1.34) | 0.88 (0.59,1.21) | 1 (1,1) |
| Neurodevelopmental composite | 24/107 (22%) | 26/107 (24%) | 0.92 (0.57,1.5) | 0.96 (0.6,1.55) | 0.9 (0.48,1.55) | 1 (0.97,1.01) |
| Cardiovascular composite | 12/107 (11%) | 14/107 (13%) | 0.86 (0.42,1.77) | 0.86 (0.42,1.77) | 0.86 (0.41,1.77) | 0.85 (0.4,1.76) |
| Cardiovascular disease risk factors |  |  | 0.94 (0.53,1.64) | 0.92 (0.52,1.64) | 0.94 (0.53,1.64)* | 0.93 (0.53,1.63) |
| 0 | 71/107 (66%) | 70/107 (65%) |  |  |  |  |
| 1 | 32/107 (30%) | 31/107 (29%) |  |  |  |  |
| >1 | 4/107 (3.7%) | 6/107 (5.6%) |  |  |  |  |
| Diabetes composite | 1/107 (0.9%) | 3/107 (2.8%) | 0.33 (0.04,3.15) | 0.38 (0.04,3.48) | 0.33 (0.04,3.15)* | 0.41 (0.04,3.86)* |
| Mental health composite | 34/107 (32%) | 38/107 (36%) | 0.89 (0.61,1.3) | 0.9 (0.62,1.32) | 0.89 (0.56,1.33) | 0.92 (0.63,1.23) |
| Any bone disease | 10/107 (9.3%) | 8/107 (7.5%) | 1.25 (0.51,3.04) | 1.23 (0.5,2.99) | 1.25 (0.5,2.86) | 1.25 (0.5,2.87) |
| Number of fractures | 0 (0, 7) | 0 (0, 7) | -0.01 (-0.27,0.25) | 0.02 (-0.23,0.26) | -0.01 (-0.27,0.25)* | 0.03 (-0.22,0.28)* |
| Fair/poor general health | 13/104 (13%) | 16/106 (15%) | 0.83 (0.42,1.63) | 0.85 (0.43,1.67) | 0.83 (0.4,1.58) | 0.94 (0.66,1.16) |
| Functional difficulties |  |  | 0.81 (0.49,1.35) | 0.82 (0.49,1.37) | 0.78 (0.43,1.39) | 0.79 (0.44,1.41) |
| No disability | 39/104 (38%) | 35/106 (33%) |  |  |  |  |
| Moderate disability | 47/104 (45%) | 49/106 (46%) |  |  |  |  |
| Severe disability | 18/104 (17%) | 22/106 (21%) |  |  |  |  |
| Fair/poor oral health | 19/104 (18%) | 26/106 (25%) | 0.74 (0.44,1.26) | 0.77 (0.46,1.31) | 0.67 (0.3,1.37) | 0.97 (0.87,1.02) |
| **Tertiary outcomes** | | | | | | |
| No secondary school qualification | 14/107 (13%) | 24/107 (22%) | 0.58 (0.32,1.07) | 0.58 (0.32,1.05) | 0.46 (0.01,15.54) | 0.47 (0.01,15.78) |
| Any disciplinary action | 5/104 (4.8%) | 12/101 (12%) | 0.4 (0.15,1.11) | 0.41 (0.15,1.12) | 0.33 (0,61.66) | 0.41 (0.15,1.13)* |
| Any convictions | 3/93 (3.2%) | 1/85 (1.2%) | 2.74 (0.29,25.86) | 2.66 (0.28,25) | 2.74 (0.29,25.86)* | 2.64 (0.28,24.84)* |
| Unemployment | 12/104 (12%) | 19/106 (18%) | 0.64 (0.33,1.26) | 0.62 (0.32,1.21) | 0.64 (0.33,1.26)* | 0.47 (0.02,13.83) |
| Alcohol use in the past year | 82/104 (79%) | 88/106 (83%) | 0.95 (0.83,1.08) | 0.95 (0.83,1.08) | 0.95 (0.83,1.08)* | 0.95 (0.83,1.09)* |
| Recreational drug use | 34/104 (33%) | 45/106 (42%) | 0.77 (0.54,1.1) | 0.73 (0.52,1.04) | 0.72 (0.41,1.12) | 0.57 (0.27,1.17) |
| Past/current smoking | 21/104 (20%) | 22/105 (21%) | 0.96 (0.57,1.64) | 0.95 (0.56,1.61) | 0.94 (0.05,19.25) | 0.91 (0.04,18.81) |
| Past/current smoking pack years | 0.25 (0.00, 1.75) | 1.00 (0.00, 6.00) | -1.48 (-2.23,-0.74) | -1.5 (-2.25,-0.76) | -1.48 (-2.23,-0.74)* | -1.5 (-2.25,-0.76)* |
| Past/current vaping | 27/104 (26%) | 37/106 (35%) | 0.74 (0.49,1.13) | 0.73 (0.49,1.1) | 0.61 (0.04,9.48) | 0.61 (0.04,9.64) |
| Components of neurodevelopmental disability composite outcome | | | | | | |
| Visual impairment | 4/104 (3.8%) | 6/106 (5.7%) | 0.68 (0.2,2.34) | 0.75 (0.22,2.55) | 0.68 (0.2,2.34)* | 0.75 (0.22,2.56)* |
| Hearing impairment | 0/107 (0%) | 4/107 (3.7%) |  |  |  |  |
| Intellectual impairment | 4/105 (3.8%) | 5/102 (4.9%) | 0.78 (0.21,2.81) | 0.83 (0.23,2.98) | 0.78 (0.21,2.81)* | 0.81 (0.22,2.95)* |
| Cerebral palsy | 6/104 (5.8%) | 2/102 (2.0%) | 2.94 (0.61,14.24) | 2.96 (0.61,14.38) | 2.94 (0.61,12.09) | 2.96 (0.6,12.69) |
| Epilepsy | 5/104 (4.8%) | 6/102 (5.9%) | 0.82 (0.26,2.59) | 0.81 (0.25,2.58) | 0.82 (0.25,2.48) | 0.8 (0.24,2.61) |
| Autism spectrum disorder | 7/107 (6.5%) | 3/105 (2.9%) | 2.29 (0.61,8.62) | 2.52 (0.67,9.49) | 2.29 (0.61,8.62)* | 1.07 (0.94,1.11) |
| Attention deficit hyperactivity disorder | 11/107 (10%) | 13/105 (12%) | 0.83 (0.39,1.77) | 0.91 (0.44,1.91) | 0.93 (0.01,77.06) | 0.98 (0.01,67.93) |
| Components of cardiovascular composite outcome | | | | | | |
| Hypertension | 9/107 (8.4%) | 12/107 (11%) | 0.75 (0.33,1.71) | 0.77 (0.34,1.75) | 0.75 (0.32,1.66) | 0.82 (0.4,1.4) |
| Cardiomyopathies | 1/104 (1.0%) | 0/102 (0%) |  |  |  |  |
| Arrhythmias | 5/104 (4.8%) | 3/102 (2.9%) | 1.63 (0.4,6.66) | 1.58 (0.39,6.41) | 1.63 (0.39,6.06) | 1.6 (0.37,6.88) |
| Heart failure | 1/107 (0.9%) | 0/107 (0%) |  |  |  |  |
| Components of cardiovascular disease risk factors composite outcome | | | | | | |
| Dyslipidaemia | 6/107 (5.6%) | 7/107 (6.5%) | 0.86 (0.3,2.47) | 0.82 (0.29,2.37) | 0.86 (0.29,2.36) | 0.82 (0.27,2.53) |
| Diabetes mellitus composite | 1/107 (0.9%) | 3/107 (2.8%) | 0.33 (0.04,3.15) | 0.38 (0.04,3.48) | 0.33 (0.04,3.15)* | 0.4 (0,25.07) |
| Overweight/obesity | 25/85 (29%) | 25/74 (34%) | 0.87 (0.55,1.38) | 0.95 (0.62,1.46) | 0.87 (0.49,1.39) | 0.86 (0.47,1.42) |
| Components of diabetes composite outcome | | | | | | |
| Prediabetes | 1/103 (1.0%) | 0/95 (0%) |  |  |  |  |
| Diabetes mellitus | 0/107 (0%) | 1/107 (0.9%) |  |  |  |  |
| Gestational diabetes mellitus | 0/8 (0%) | 2/3 (67%) |  |  |  |  |
| Components of mental health composite outcome | | | | | | |
| Depression | 28/105 (27%) | 22/106 (21%) | 1.28 (0.79,2.09) | 1.3 (0.8,2.11) | 1.3 (0.76,2.05) | 1.28 (0.77,1.93) |
| Bipolar affective disorder | 1/106 (0.9%) | 0/107 (0%) |  |  |  |  |
| Anxiety disorders | 28/106 (26%) | 28/106 (26%) | 1 (0.64,1.57) | 0.99 (0.63,1.55) | 1.03 (0.59,1.65) | 1.02 (0.53,1.87) |
| Suicide/self-harm | 1/102 (1.0%) | 0/94 (0%) |  |  |  |  |
| Components of functional difficulties outcomes | | | | | | |
| Difficulty seeing, even if wearing glasses |  |  | 1.1 (0.59,2.08) | 1.13 (0.6,2.14) | 1.1 (0.59,2.08)* | 1.13 (0.6,2.13)* |
| No disability | 78/104 (75%) | 82/106 (77%) |  |  |  |  |
| Moderate disability | 22/104 (21%) | 18/106 (17%) |  |  |  |  |
| Severe disability | 4/104 (3.8%) | 6/106 (5.7%) |  |  |  |  |
| Difficulty hearing, even if using a hearing aid |  |  | 1.12 (0.42,3.03) | 1.13 (0.42,3.07) | 1.12 (0.42,3.03)* | 1.25 (0.03,61.57) |
| No disability | 95/104 (91%) | 98/106 (92%) |  |  |  |  |
| Moderate disability | 9/104 (8.7%) | 5/106 (4.7%) |  |  |  |  |
| Severe disability | 0/104 (0%) | 3/106 (2.8%) |  |  |  |  |
| Difficulty walking or climbing steps |  |  | 1.28 (0.63,2.59) | 1.29 (0.63,2.62) | 1.28 (0.63,2.59)* | 1.23 (0.03,45.22) |
| No disability | 83/104 (80%) | 89/106 (84%) |  |  |  |  |
| Moderate disability | 18/104 (17%) | 12/106 (11%) |  |  |  |  |
| Severe disability | 3/104 (2.9%) | 5/106 (4.7%) |  |  |  |  |
| Difficulty remembering or concentrating |  |  | 0.86 (0.51,1.45) | 0.86 (0.5,1.46) | 0.86 (0.51,1.46) | 0.86 (0.51,1.47) |
| No disability | 56/103 (54%) | 53/106 (50%) |  |  |  |  |
| Moderate disability | 34/103 (33%) | 39/106 (37%) |  |  |  |  |
| Severe disability | 13/103 (13%) | 14/106 (13%) |  |  |  |  |
| Difficulty washing all over or dressing |  |  | 0.74 (0.23,2.4) | 0.7 (0.21,2.31) | 0.74 (0.23,2.4)* | 0.7 (0.21,2.3)* |
| No disability | 99/104 (95%) | 99/106 (93%) |  |  |  |  |
| Moderate disability | 1/104 (1.0%) | 5/106 (4.7%) |  |  |  |  |
| Severe disability | 4/104 (3.8%) | 2/106 (1.9%) |  |  |  |  |
| Difficulty communicating |  |  | 1.04 (0.56,1.92) | 1.06 (0.57,1.98) | 1.04 (0.52,2.08) | 1.08 (0.55,2.13) |
| No disability | 77/104 (74%) | 79/106 (75%) |  |  |  |  |
| Moderate disability | 21/104 (20%) | 22/106 (21%) |  |  |  |  |
| Severe disability | 6/104 (5.8%) | 5/106 (4.7%) |  |  |  |  |
| Abbreviations: CI, confidence interval; GLM, generalized linear models; GLMM, generalized linear mixed models.  Data are n (%) or median (maximum, minimum).  ^a^ Relative risk provided for binary outcomes, proportional odds ratios for categorical ordinal outcomes or mean difference for counts.  ^b^ Adjusted for gestational age at randomisation and multiplicity (yes/no).  ^c^ GLMMs account for clustering due to multiple births except where indicated by *.  ^d^ Adjusted for gestational age at randomisation. | | | | | | |
