# Supplementary material for: Twenty-year outcomes after repeat doses of antenatal corticosteroids prior to 32 weeks’ gestation: Follow-up of a randomised clinical trial
Source: PLoS Med. 2025 May 28;22(5):e1004618. doi: 10.1371/journal.pmed.1004618 (PMC12118977; doi:10.1371/journal.pmed.1004618)
Supplement: S2 Table — (DOCX) [file pmed.1004618.s003.docx]

S2 Table. Subgroup analyses: sex

| **Outcome** | **Repeat** | **Placebo** | **Unadjusted effect (95% CI)^a^** | **Adjusted effect (95% CI)^a,b^** | **Interaction p-value** |
| --- | --- | --- | --- | --- | --- |
| **Primary outcome** |  |  |  |  |  |
| Any asthma |  |  |  |  | 0.06 |
| Male | 39/64 (61%) | 23/54 (43%) | 1.43 (0.99,2.06) | 1.43 (1.00,2.03) |  |
| Female | 19/43 (44%) | 27/53 (51%) | 0.87 (0.57,1.33) | 0.86 (0.56,1.32) |  |
| **Secondary outcomes** |  |  |  |  |  |
| Asthma currently on treatment |  |  |  |  | 0.74 |
| Male | 20/64 (31%) | 16/54 (30%) | 1.05 (0.61,1.83) | 1.03 (0.61,1.76) |  |
| Female | 12/43 (28%) | 17/53 (32%) | 0.87 (0.47,1.62) | 0.91 (0.49,1.67) |  |
| Death (any cause after randomisation) |  |  |  |  | 0.11 |
| Male | 3/92 (3.3%) | 2/95 (2.1%) | 1.55 (0.26,9.06) | 2.07 (0.35,12.38) |  |
| Female | 1/83 (1.2%) | 5/82 (6.1%) | 0.2 (0.02,1.65) | 0.2 (0.02,1.67) |  |
| Respiratory composite |  |  |  |  | 0.68 |
| Male | 26/62 (42%) | 27/53 (51%) | 0.82 (0.55,1.22) | 0.98* |  |
| Female | 14/43 (33%) | 17/49 (35%) | 0.94 (0.53,1.67) | 0.96 (0.54,1.69) |  |
| Neurodevelopmental composite |  |  |  |  | 0.52 |
| Male | 16/64 (25%) | 17/54 (31%) | 0.79 (0.45,1.42) | 0.84 (0.48,1.48) |  |
| Female | 8/43 (19%) | 9/53 (17%) | 1.1 (0.46,2.6) | 1.13 (0.48,2.67) |  |
| Cardiovascular composite |  |  |  |  | 0.08 |
| Male | 5/64 (7.8%) | 9/54 (17%) | 0.47 (0.17,1.31) | 0.48 (0.17,1.34) |  |
| Female | 7/43 (16%) | 5/53 (9.4%) | 1.73 (0.59,5.06) | 1.69 (0.58,4.93) |  |
| Cardiovascular disease risk factors |  |  |  |  | 0.99 |
| Male |  |  | 0.92 (0.43,1.96) | 0.93 (0.43,2.04) |  |
| 0 | 43/64 (67%) | 35/54 (65%) |  |  |  |
| 1 | 18/64 (28%) | 17/54 (31%) |  |  |  |
| >1 | 3/64 (4.7%) | 2/54 (3.7%) |  |  |  |
| Female |  |  | 0.96 (0.42,2.22) | 0.93 (0.4,2.16) |  |
| 0 | 28/43 (65%) | 35/53 (66%) |  |  |  |
| 1 | 14/43 (33%) | 14/53 (26%) |  |  |  |
| >1 | 1/43 (2.3%) | 4/53 (7.5%) |  |  |  |
| Diabetes composite |  |  |  |  | 1.00 |
| Male | 1/64 (1.6%) | 0/54 (0%) |  |  |  |
| Female | 0/43 (0%) | 3/53 (5.7%) |  |  |  |
| Mental health composite |  |  |  |  | 0.43 |
| Male | 14/64 (22%) | 15/54 (28%) | 0.79 (0.42,1.48) | 0.81 (0.43,1.53) |  |
| Female | 20/43 (47%) | 23/53 (43%) | 1.07 (0.69,1.67) | 1.07 (0.69,1.67) |  |
| Any bone disease |  |  |  |  | 0.21 |
| Male | 3/64 (4.7%) | 4/54 (7.4%) | 0.63 (0.15,2.7) | 0.63 (0.15,2.66) |  |
| Female | 7/43 (16%) | 4/53 (7.5%) | 2.16 (0.68,6.89) | 2.13 (0.67,6.82) |  |
| Number of fractures |  |  |  |  | 0.40 |
| Male | 0 (0, 5) | 0 (0, 4) | 0.17 (-0.14,0.49) | 0.09 (-0.2,0.37) |  |
| Female | 0 (0, 7) | 1 (0, 7) | -0.16 (-0.59,0.26) | -0.13 (-0.55,0.29) |  |
| Fair/poor general health |  |  |  |  | 0.99 |
| Male | 7/62 (11%) | 7/53 (13%) | 0.85 (0.32,2.28) | 0.89 (0.34,2.34) |  |
| Female | 6/42 (14%) | 9/53 (17%) | 0.84 (0.33,2.18) | 0.83 (0.32,2.15) |  |
| Functional difficulties |  |  |  |  | 0.08 |
| Male |  |  | 0.57 (0.28,1.15) | 0.57 (0.28,1.17) |  |
| No disability | 28/62 (45%) | 19/53 (36%) |  |  |  |
| Moderate disability | 28/62 (45%) | 22/53 (42%) |  |  |  |
| Severe disability | 6/62 (9.7%) | 12/53 (23%) |  |  |  |
| Female |  |  | 1.43 (0.66,3.07) | 1.46 (0.67,3.16) |  |
| No disability | 11/42 (26%) | 16/53 (30%) |  |  |  |
| Moderate disability | 19/42 (45%) | 27/53 (51%) |  |  |  |
| Severe disability | 12/42 (29%) | 10/53 (19%) |  |  |  |
| Fair/poor oral health |  |  |  |  | 0.01 |
| Male | 8/62 (13%) | 18/53 (34%) | 0.38 (0.18,0.8) | 0.39 (0.19,0.82) |  |
| Female | 11/42 (26%) | 8/53 (15%) | 1.74 (0.77,3.92) | 1.76 (0.78,3.98) |  |
| Abbreviations: CI, confidence interval.  Data are n/N (%) or median (minimum, maximum).  ^a^ Relative risk provided for binary outcomes, proportional odds ratios for categorical ordinal outcomes or mean difference for counts.  ^b^ Adjusted for gestational age at randomization and multiplicity.  * Confidence intervals for relative risk not calculable. | | | | | |
