# Supplementary material for: Twenty-year outcomes after repeat doses of antenatal corticosteroids prior to 32 weeks’ gestation: Follow-up of a randomised clinical trial
Source: PLoS Med. 2025 May 28;22(5):e1004618. doi: 10.1371/journal.pmed.1004618 (PMC12118977; doi:10.1371/journal.pmed.1004618)
Supplement: S3 Table — (DOCX) [file pmed.1004618.s004.docx]

S3 Table. Subgroup analyses: deprivation

| **Outcome** | **Repeat** | **Placebo** | **Unadjusted effect (95% CI)^a^** | **Adjusted effect (95% CI)^a,b^** | **Interaction p-value** |
| --- | --- | --- | --- | --- | --- |
| **Primary outcome** |  |  |  |  |  |
| Any asthma |  |  |  |  | 0.09 |
| Most deprived | 21/31 (68%) | 15/33 (45%) | 1.49 (0.95,2.33) | 1.44 (0.95,2.18) |  |
| Less deprived | 37/74 (50%) | 34/70 (49%) | 1.03 (0.74,1.43) | 1.06 (0.77,1.46) |  |
| **Secondary outcomes** |  |  |  |  |  |
| Asthma currently on treatment |  |  |  |  | 0.17 |
| Most deprived | 12/31 (39%) | 10/33 (30%) | 1.28 (0.65,2.52) | 1.55 (0.83,2.9) |  |
| Less deprived | 20/74 (27%) | 22/70 (31%) | 0.86 (0.52,1.43) | 0.85 (0.51,1.4) |  |
| Respiratory composite |  |  |  |  | 0.18 |
| Most deprived | 9/31 (29%) | 16/32 (50%) | 0.58 (0.3,1.11) | 0.63 (0.33,1.22) |  |
| Less deprived | 31/72 (43%) | 25/66 (38%) | 1.14 (0.76,1.71) | 1.27 (0.89,1.8) |  |
| Neurodevelopmental composite |  |  |  |  | 0.20 |
| Most deprived | 9/31 (29%) | 8/33 (24%) | 1.2 (0.53,2.71) | 1.25 (0.61,2.56) |  |
| Less deprived | 14/74 (19%) | 18/70 (26%) | 0.74 (0.4,1.36) | 0.72 (0.39,1.32) |  |
| Cardiovascular composite |  |  |  |  | 0.58 |
| Most deprived | 6/31 (19%) | 6/33 (18%) | 1.06 (0.38,2.95) | 1.22 (0.43,3.42) |  |
| Less deprived | 6/74 (8.1%) | 8/70 (11%) | 0.71 (0.26,1.94) | 0.71 (0.26,1.95) |  |
| Cardiovascular disease risk factors |  |  |  |  | 0.68 |
| Most deprived |  |  | 1.06 (0.4,2.78) | 1.13 (0.4,3.18) |  |
| 0 | 16/31 (52%) | 18/33 (55%) |  |  |  |
| 1 | 14/31 (45%) | 13/33 (39%) |  |  |  |
| >1 | 1/31 (3.2%) | 2/33 (6.1%) |  |  |  |
| Less deprived |  |  | 0.91 (0.45,1.85) | 0.87 (0.42,1.79) |  |
| 0 | 53/74 (72%) | 49/70 (70%) |  |  |  |
| 1 | 18/74 (24%) | 17/70 (24%) |  |  |  |
| >1 | 3/74 (4.1%) | 4/70 (5.7%) |  |  |  |
| Diabetes composite |  |  |  |  | 1.00 |
| Most deprived | 0/31 (0%) | 1/33 (3.0%) |  |  |  |
| Less deprived | 1/74 (1.4%) | 2/70 (2.9%) | 0.47 (0.04,5.1) | 0.48 (0.05,4.86) |  |
| Mental health composite |  |  |  |  | 0.10 |
| Most deprived | 13/31 (42%) | 10/33 (30%) | 1.38 (0.71,2.69) | 1.64 (NaN,NaN)* |  |
| Less deprived | 21/74 (28%) | 27/70 (39%) | 0.74 (0.46,1.17) | 0.73 (0.46,1.16) |  |
| Any bone disease |  |  |  |  | 0.30 |
| Most deprived | 4/31 (13%) | 2/33 (6.1%) | 2.13 (0.42,10.81) | 2.2 (0.42,11.38) |  |
| Less deprived | 5/74 (6.8%) | 6/70 (8.6%) | 0.79 (0.25,2.47) | 0.74 (0.24,2.32) |  |
| Number of fractures |  |  |  |  | 0.45 |
| Most deprived | 0 (0, 5) | 0 (0, 5) | -0.11 (-0.57,0.35) | -0.13 (-0.55,0.29) |  |
| Less deprived | 0 (0, 7) | 0 (0, 7) | -0.01 (-0.34,0.31) | 0.09 (-0.21,0.38) |  |
| Fair/poor general health |  |  |  |  | 0.98 |
| Most deprived | 5/29 (17%) | 7/33 (21%) | 0.81 (0.29,2.28) | 0.77 (0.27,2.18) |  |
| Less deprived | 8/73 (11%) | 9/70 (13%) | 0.85 (0.35,2.08) | 0.94 (0.39,2.27) |  |
| Functional difficulties |  |  |  |  | 0.36 |
| Most deprived |  |  | 1.08 (0.42,2.77) | 1.5 (0.56,4.02) |  |
| No disability | 7/29 (24%) | 10/33 (30%) |  |  |  |
| Moderate disability | 16/29 (55%) | 15/33 (45%) |  |  |  |
| Severe disability | 6/29 (21%) | 8/33 (24%) |  |  |  |
| Less deprived |  |  | 0.76 (0.41,1.41) | 0.71 (0.38,1.33) |  |
| No disability | 31/73 (42%) | 25/70 (36%) |  |  |  |
| Moderate disability | 30/73 (41%) | 31/70 (44%) |  |  |  |
| Severe disability | 12/73 (16%) | 14/70 (20%) |  |  |  |
| Fair/poor oral health |  |  |  |  | 0.67 |
| Most deprived | 6/29 (21%) | 8/33 (24%) | 0.85 (0.34,2.17) | 0.99 (0.39,2.47) |  |
| Less deprived | 13/73 (18%) | 17/70 (24%) | 0.73 (0.39,1.4) | 0.78 (0.41,1.48) |  |
| Abbreviations: CI, confidence interval.  Data are n/N (%) or median (minimum, maximum).  ^a^ Relative risk provided for binary outcomes, proportional odds ratios for categorical ordinal outcomes or mean difference for counts.  ^b^ Adjusted for gestational age at randomization and multiplicity.  * Confidence intervals for relative risk not calculable. | | | | | |
