# Supplementary material for: Twenty-year outcomes after repeat doses of antenatal corticosteroids prior to 32 weeks’ gestation: Follow-up of a randomised clinical trial
Source: PLoS Med. 2025 May 28;22(5):e1004618. doi: 10.1371/journal.pmed.1004618 (PMC12118977; doi:10.1371/journal.pmed.1004618)
Supplement: S4 Table — (DOCX) [file pmed.1004618.s005.docx]

S4 Table. Sensitivity analyses

| **Outcome** | **Repeat** | **Placebo** | **Unadjusted RR (95% CI)** | **Adjusted RR (95% CI)** |
| --- | --- | --- | --- | --- |
| Any asthma | 58/107 (54%) | 50/107 (47%) | 1.16 (0.89,1.51) | 1.16 (0.89,1.5)^a^ |
| Asthma >5 years and currently on treatment | 24/107 (22%) | 29/107 (27%) | 0.83 (0.52,1.32) | 0.81 (0.51,1.29)^b^ |
| Respiratory primary diagnosis composite | 35/105 (33%) | 34/102 (33%) | 1 (0.68,1.47) | 1.06 (0.74,1.52)^b^ |
| Abbreviations: CI, confidence interval.  Data are n/N (%).  ^a^ Adjusted for gestational age at randomisation, multiplicity, birth centre and maternal smoking at first antenatal visit.  ^b^ Adjusted for gestational age at randomisation, multiplicity and birth centre. | | | | |
