# Supplementary material for: Twenty-year outcomes after repeat doses of antenatal corticosteroids prior to 32 weeks’ gestation: Follow-up of a randomised clinical trial
Source: PLoS Med. 2025 May 28;22(5):e1004618. doi: 10.1371/journal.pmed.1004618 (PMC12118977; doi:10.1371/journal.pmed.1004618)
Supplement: S5 Table — (DOCX) [file pmed.1004618.s006.docx]

S5 Table. Enriched sample analysis

| **Outcome** | **Repeat** | **Placebo** | **Unadjusted RR (95% CI)** | **Adjusted RR (95% CI)^a^** |
| --- | --- | --- | --- | --- |
| Any asthma | 83/158 (53%) | 80/165 (48%) | 1.08 (0.87,1.34) | 1.07 (0.84,1.36) |
| Visual impairment | 4/159 (2.5%) | 6/166 (3.6%) | 0.7 (0.2,2.42) | 0.74 (0.22,2.53) |
| Hearing impairment | 1/159 (0.6%) | 6/166 (3.6%) | 0.17 (0.02,1.43) | 0.25 (0.03,2.21) |
| Cerebral palsy | 7/130 (5.4%) | 3/126 (2.4%) | 2.26 (0.6,8.55) | 3.58 (0.76,16.88) |
| Intellectual impairment | 7/161 (4.3%) | 6/167 (3.6%) | 1.21 (0.42,3.52) | 1.28 (0.4,4.07) |
| Abbreviations: CI, confidence interval.  Data are n/N (%).  ^a^ Adjusted for gestational age at randomisation, multiplicity and birth centre. | | | | |
