# Supplementary figures and images for: Twenty-year outcomes after repeat doses of antenatal corticosteroids prior to 32 weeks’ gestation: Follow-up of a randomised clinical trial
Source: PLoS Med. 2025 May 28;22(5):e1004618. doi: 10.1371/journal.pmed.1004618 (PMC12118977; doi:10.1371/journal.pmed.1004618)

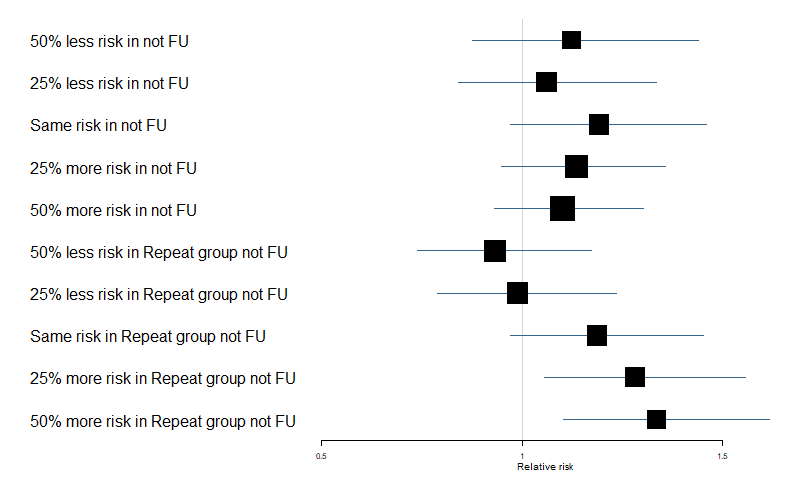

Supplement: S1 Fig — (TIF) [file pmed.1004618.s007.tif]
