# Supplementary material for: Twenty-year outcomes after repeat doses of antenatal corticosteroids prior to 32 weeks’ gestation: Follow-up of a randomised clinical trial
Source: PLoS Med. 2025 May 28;22(5):e1004618. doi: 10.1371/journal.pmed.1004618 (PMC12118977; doi:10.1371/journal.pmed.1004618)
Supplement: S1 Text — (DOCX) [file pmed.1004618.s008.docx]

S1 Text. Supplementary methods for missing data sensitivity analysis.

To assess the assumption of missingness at random where participants not lost to follow-up are exchangeable with those lost to follow-up, multivariate imputation by chained equations was performed using the mice package [1]. This method creates replacement values for missing data based on fully conditional specification, where each incomplete variable is imputed by a separate model. The logistic regression method was used to impute the binary primary outcome of any asthma diagnosis based on the available participant characteristics in Table 2. Five imputed datasets were created and estimates were pooled across imputations.

To assess the assumption of missingness not at random, a pattern-mixture modeling approach was used [2]. First, to investigate the impact if missingness was related to loss to follow-up, for participants not followed up, the baseline probability of any asthma diagnosis was assumed to be the same as those followed up or increased or decreased by 25% and 50% on the odds scale. The adjusted probabilities were bounded between 0 and 1 to ensure validity, and new outcomes were randomly drawn from a Bernoulli distribution using these adjusted probabilities. Next, to investigate the impact if missingness was related to both loss to follow-up and exposure to repeat antenatal corticosteroids, the probability of the outcome for those not followed up in the placebo group was set to be the same as that of cohort, while for participants not followed up in the repeat group, this baseline probability was assumed to be the same as those followed up or increased or decreased by 25% and 50% on the odds scale. Once again, the adjusted probabilities were bounded between 0 and 1 to ensure validity, and new outcomes were randomly drawn from a Bernoulli distribution using these adjusted probabilities. Following these modifications, the analysis was repeated as per the primary analysis.

## References

1. van Buuren S, Groothuis-Oudshoorn K. mice: Multivariate Imputation by Chained Equations in R. Journal of Statistical Software. 2011;45(3):1-67. doi: 10.18637/jss.v045.i03.

2. Khalifa A, Graf GHJ. A practical guide to sensitivity analysis for causal effects in the presence of non-ignorable loss to follow-up 2022. Available from: <https://bookdown.org/glorya_hu/MNAR-Guide/>.
