# Supplementary material for: Twenty-year outcomes after repeat doses of antenatal corticosteroids prior to 32 weeks’ gestation: Follow-up of a randomised clinical trial
Source: PLoS Med. 2025 May 28;22(5):e1004618. doi: 10.1371/journal.pmed.1004618 (PMC12118977; doi:10.1371/journal.pmed.1004618)
Supplement: S1 Statistical Analysis Plan — (PDF) [file pmed.1004618.s009.pdf]

The AnteNatal Corticosteroids Health Outcomes Review (ANCHOR) Study: Australasian Collaborative Trial of Repeat Doses of Steroids (ACTORDS) follow-up

Statistical Analysis Plan

Version 1.6, 31 July 2024

SAP Author: Robyn May

Approved by	Signature	Date
Dist Prof Dame Jane Harding (Principal Investigator)		
Greg Gamble (Statistician)		

The AnteNatal Corticosteroids Health Outcomes Review (ANCHOR) Study: Australasian Collaborative Trial of Repeat Doses of Steroids (ACTORDS) follow-up

Statistical Analysis Plan

Version 1.4, 19 April 2024

SAP Author: Robyn May

Approved by	Signature	Date
Dist Prof Dame Jane Harding (Principal Investigator)	[signature]	14/5/24
Greg Gamble (Statistician)	[signature]	10/05/24

Contents

1. Scope 4
2. Study information 4
   Aim 4
   Study hypothesis 4
3. Background: The Australasian Collaborative Trial of Repeat Doses of Steroids (ACTORDS) 4
   ACTORDS RCT 4
   2-year follow-up 4
   6–8-year follow-up 5
   In-depth 6–8-year follow-up of the New Zealand ACTORDS cohort 5
4. Methods for the ANCHOR Study: follow-up of ACTORDS F1 6
   Study design 6
   Inclusion Criteria 6
   Exclusion criteria 6
5. Study outcomes 6
6. Outcome reporting 8
7. Steering Group 8
8. Power calculation 8
9. Analysis based on Self-reported Outcomes 8
10. General issues for statistical analysis 8
   Analysis software 8
   Analysis approach 9
   Approach for withdrawals, missing data and outliers 9
   Protocol deviations 9
   Covariates and potential confounders 9
   Multiple comparisons and multiplicity 9
   Clustering 9
11. Descriptive analysis 9
12. Statistical Analysis 9
13. Exploratory analyses 10
   Enriched Sample Analysis 10
   Subgroup analyses 10
   Sensitivity analyses 10
   Post hoc analyses 10
14. References 10

15. Appendix 11
15.1. Flow diagram 11
15.2. Outcomes list 13
Primary outcome 15
Secondary outcomes 16
Tertiary Outcomes 39

1. Scope

This Statistical Analysis Plan (SAP) describes the outcomes and analyses for the long term follow-up of the Australasian Collaborative Trial of Repeat Doses of Steroids (ACTORDS). In this plan the mothers who participated in ACTORDS are referred to as the F0 generation and their children the F1 generation. This SAP concerns the twenty-year follow-up of children (F1 generation) of the participants of ACTORDS. This follow-up study was conducted as part of the AnteNatal Corticosteroids Health Outcomes Review (ANCHOR) Study.

2. Study information

Aim

To determine if repeat antenatal corticosteroids, given to women at risk of preterm birth, have long term impacts on the health of offspring (F1).

Study hypothesis

Repeat antenatal corticosteroids reduce neonatal mortality and morbidity without long-term adverse impacts on health and social outcomes in offspring (F1).

3. Background: The Australasian Collaborative Trial of Repeat Doses of Steroids (ACTORDS)

ACTORDS RCT[1]

In this multicentre centre, two-arm, parallel, randomised double blind placebo-controlled trial, women who remained at risk of preterm birth at less than 32 weeks' gestation, 7 or more days after receiving a first course of prenatal corticosteroids, were randomly assigned to receive a repeat intramuscular dose of either betamethasone or saline placebo. This could be repeated every week the woman remained undelivered, at less than 32 weeks' gestation, if still considered to be at risk of preterm birth in the next 7 days. Primary outcomes were occurrence and severity of neonatal respiratory distress syndrome, severity of any lung disease present, use and duration of oxygen and mechanical ventilation, and weight, length, and head circumference at birth and hospital discharge.

The RCT found that fewer babies exposed to repeat corticosteroids had respiratory distress syndrome and fewer had severe lung disease than those in the placebo group. In keeping with these benefits, babies exposed to repeat corticosteroids needed less oxygen therapy, and shorter duration of mechanical ventilation. Mean weight, length, and head circumference at birth and hospital discharge did not differ between treatment groups. Z-scores for weight and head circumference at birth were lower in the babies who received repeat corticosteroids although at the time of hospital discharge Z-scores did not differ between treatment groups.

2-year follow-up[2]

The primary outcome of the 2-year follow-up study was survival free of major neurosensory disability and body size of the children at 2 years of corrected age.

Of the 1085 children who were alive at 2 years of age, 1047 (96.5%) were seen for assessment (521 exposed to repeat-corticosteroid treatment and 526 exposed to placebo). The rate of survival free of

major disability was similar in the repeat corticosteroid and placebo groups (84.4% and 81.0%, respectively; adjusted relative risk, 1.04, 95% confidence interval, 0.98 to 1.10; adjusted P = 0.20). There were no significant differences between the groups in body size, blood pressure, use of health services, respiratory morbidity, or child behaviour scores, although children exposed to repeat doses of corticosteroids were more likely than those exposed to placebo to warrant assessment for attention problems (P = 0.04).

6–8-year follow-up[3, 4]

The primary outcome of the mid-childhood follow-up study was survival free of neurosensory disability.

Of the 1059 eligible long-term survivors, 963 (91%) were included in the primary outcome; 479 (91%) in the repeat corticosteroid group and 484 (91%) in the placebo group. The rate of survival free of neurosensory disability was similar in both groups (78.3% repeat versus 77.3% placebo; risk ratio 1.00, 95% confidence interval, 0.94–1.08). Neurodevelopment, including cognitive function, and behaviour, body size, blood pressure, spirometry, and health-related quality of life, were similar in both groups, as was the use of health services.

A secondary analysis was also undertaken to determine the influence of fetal growth restriction (FGR) on the effects of repeated doses of antenatal betamethasone on neurocognitive function in mid-childhood. The FGR rate was 139 of 493 children (28.2%) in the repeat betamethasone treatment group and 122 of 495 (24.6%) in the placebo group. The rates of the primary outcomes, survival free of any disability and death or moderate to severe disability, were similar between treatment groups in both the FGR and non-FGR subgroups, with no evidence that FGR influenced treatment effect.

In-depth 6–8-year follow-up of the New Zealand ACTORDS cohort[5, 6]

In the New Zealand ACTORDS cohort, an extended follow-up was undertaken to investigate the long-term effects of repeat antenatal betamethasone treatment on risk factors for cardiovascular and metabolic disease and bone health. The children had to be living in New Zealand to be eligible, so this cohort included some children randomised in Australia but living in New Zealand, and excluded some randomised in New Zealand but living elsewhere.

Of 320 eligible childhood survivors for the study of cardiometabolic risk factors, 258 were assessed (81%; 123 repeat betamethasone group; 135 placebo [single course] group). Children exposed to repeat antenatal betamethasone and those exposed to placebo had similar whole-body and regional fat and lean mass; insulin sensitivity, acute insulin secretion and fasting glucose and insulin concentrations; ambulatory blood pressure and heart rate, including nocturnal; and estimated glomerular filtration rate.

Of 212 childhood survivors eligible for whole-body dual-energy radiograph absorptiometry to assess bone mass, 185 were studied (87%; 91 repeat betamethasone group; 94 placebo [single course] group). Children exposed to repeat antenatal betamethasone and those exposed to placebo had similar whole-body and spinal bone mineral content and bone area, and risk of fractures.

4. Methods for the ANCHOR Study: follow-up of ACTORDS F1

Study design

This study consists of long term follow-up of ACTORDS at 20 years comparing outcomes in offspring (F1) exposed to repeat prenatal betamethasone to those exposed to placebo (single course of corticosteroids).

Follow-up consisted of a questionnaire based on the New Zealand Health Survey and consent to accessing routinely collected data from government agencies including the Ministry of Health, Testsafe, Ministry of Education, New Zealand Qualifications Authority, Accident Compensation Corporation, Ministry of Justice, Whaikaha (Ministry of Disabled People) and Statistics New Zealand.

Inclusion Criteria

Participants in this follow-up study were eligible for inclusion if they had been born to a participant in ACTORDS who was randomised in New Zealand and written informed consent was obtained. Participation in the 2-year follow-up study or the 6–8-year follow-up study was not a requirement for participation.

Exclusion criteria

Declined participation or unable to obtain informed consent.

5. Study outcomes

Outcome definitions and the hierarchical approach to different data sources are listed below and detailed in the appendix. All components of the composite secondary outcomes will be reported separately as tertiary outcomes. Administrative data will be right censored at the date of the questionnaire for participants who completed the questionnaire and at the date of consent for those who did not.

Primary Outcome

- Any asthma diagnosis

Secondary Outcomes:

- Asthma currently on treatment
- Death (any cause after randomisation)
- Respiratory outcomes (composite) – any respiratory diagnoses other than asthma including chronic lung disease
- Neurodevelopmental disability (composite) – any of:
  - visual impairment
  - hearing impairment
  - intellectual impairment
  - cerebral palsy
  - epilepsy
  - autism spectrum disorder (including Asperger's syndrome)
  - attention deficit disorder (ADD) or attention deficit hyperactivity disorder (ADHD)
- Cardiovascular outcomes (composite) – any cardiovascular diagnosis including hypertension, cardiomyopathies, arrhythmias, heart failure but excluding congenital heart disease
- Number of cardiovascular disease risk factors (composite categorised as 0, 1 or >1 risk factors) – any of:
  - high blood pressure
  - dyslipidaemia

    - diabetes mellitus
    - obesity/overweight
- Diabetes outcomes (composite) – any of prediabetes, diabetes mellitus, gestational diabetes mellitus
- Mental health outcomes (composite) – any mental health diagnosis including depression, bipolar affective disorder, schizophrenia, anxiety disorders, suicide/self-harm
- Bone health outcomes: any diagnoses related to bone disease
- Bone health outcomes: number of fractures
- General health outcomes: self-reported general health (reported health fair or poor)
- General health outcomes: functional difficulties (categorised as no disability / moderate or non-severe disabilities / severe disabilities)
- General health outcomes: oral health (reported oral health fair or poor or tooth extractions)

Tertiary Outcomes:

- Educational achievement outcomes: no secondary school qualification
- Educational achievement outcomes: any disciplinary action
- Social outcomes: any convictions
- Social outcomes: unemployment
- Social outcomes: alcohol use in the past year
- Social outcomes: recreational drug use
- Social outcomes: past/current smoking (pack years)
- Social outcomes: past/current vaping
- Components of neurodevelopmental disability composite outcome:
    - visual impairment
    - hearing impairment
    - intellectual impairment
    - cerebral palsy
    - epilepsy
    - autism spectrum disorder (including Asperger's syndrome)
    - attention deficit disorder (ADD) or attention deficit hyperactivity disorder (ADHD)
- Components of cardiovascular composite outcome:
    - Hypertension
    - Cardiomyopathies
    - Arrhythmias
    - heart failure
- Components of cardiovascular disease risk factors composite outcome:
    - high blood pressure
    - dyslipidaemia
    - diabetes mellitus
    - obesity/overweight
- Components of diabetes composite outcome:
    - prediabetes/diabetes mellitus
    - gestational diabetes mellitus
- Components of mental health composite outcome
    - depression
    - bipolar affective disorder
    - anxiety disorders
    - suicide/self-harm

- schizophrenia
- Components of functional difficulties outcome
  - Difficulty seeing, even if wearing glasses
  - Difficulty hearing, even if using a hearing aid
  - Difficulty walking or climbing steps
  - Difficulty remembering or concentrating
  - Difficulty washing all over or dressing
  - Difficulty communicating
- Components of obesity/overweight outcome
  - BMI
  - height
  - weight

6. Outcome reporting

Outcomes may not all be reported in the same publication, instead being grouped into categories for reporting. These categories will likely include cardiometabolic and respiratory outcomes, other health outcomes and socioeconomic outcomes.

7. Steering Group

Distinguished Professor Dame Jane Harding, Professor Stuart Dalziel, Dr Carl Eagleton, Professor Caroline Crowther, Associate Professor Barry Milne, Mr Greg Gamble, Associate Professor Chris McKinlay

8. Power calculation

This study involves the follow-up of a clinical trial, and the sample size is limited to the number of participants randomised in New Zealand and able to be located. For the primary outcome of any asthma diagnosis, the sample size of 214 allows for detection of a 22% increase in the proportion with the outcome (relative risk 1.63) with 90% power (β = 0.10) at a significance level of 5% (α = 0.05),assuming a baseline prevalence of 35% in the placebo group (as was found in the 6-8 year follow-up for the whole cohort[3]).

9. Analysis based on Self-reported Outcomes

An aim of the ANCHOR research program was to compare outcomes based on expert clinical synthesis of administrative and self-reported data and those based on questionnaire responses completed by each participant. We have found that, while there are differences in the outcomes between these two data sources in 50-year-olds, overall differences between randomised groups remained the same regardless of the data source.[7] Therefore, the synthesis of administrative and self-reported data will be used in this analysis.

10. General issues for statistical analysis

Analysis software

All analyses will be performed using R[8].

Analysis approach

Analyses will be performed using an intention-to-treat approach with participants analysed according to the initial treatment group to which their mother was allocated. The denominator for the outcome of death (any cause after randomisation) will be the total number of fetuses alive at randomisation in the NZ cohort (n=352). All other study outcomes will use the total number of participants who gave consent (n=214).

Approach for withdrawals, missing data and outliers

Data up to the point of withdrawal will be used for those participants who have withdrawn from the study unless they have withdrawn their consent for use of their data. No imputation will be performed for missing data. We do not plan to exclude outliers from the analysis.

Protocol deviations

Participants will be included in the intention-to-treat analysis, regardless of protocol deviations.

Covariates and potential confounders

Adjusted and unadjusted results will be presented for all outcomes. The results will be adjusted for:

- Gestational age at randomisation (continuous variable).

Additional baseline variables will be included in the model if these differ markedly between treatment groups and are known to influence the particular outcome.

Multiple comparisons and multiplicity

Each of the primary outcomes will be tested at the 5% significance level. No adjustment to the significance level will be made for secondary or tertiary outcomes, subgroup analyses or sensitivity analyses. The total number of tests will be reported to allow interpretation of the risk of type I error.

Clustering

As 20% of the ACTORDS cohort were multiple pregnancies, clustering will be accounted for in this analysis using generalised linear mixed-effects regression models with inclusion of the unique maternal identifier as a random effect to account for the nonindependence of children from multiple pregnancies.

11. Descriptive analysis

A flow chart will be completed to describe participant flow from randomisation to 20-year follow-up.

Summary statistics will be presented for F1 participants included in the intention-to-treat analysis, with a comparison between characteristics of those followed up and those not followed up. Baseline demographic variables will be reported as mean and standard deviation or median and interquartile range where appropriate for continuous variables and as frequency and percentages for categorical variables.

12. Statistical Analysis

Statistical analysis will be performed for all primary, secondary and tertiary outcomes. Analyses will compare the repeat antenatal corticosteroid (betamethasone) group to the placebo (single course) group.

Analyses for binary outcomes will be performed adjusted for covariates listed above using mixed-effects generalised linear models assuming a binary distribution and log link function, reported as relative risk (RR) with 95% confidence intervals.

Analyses for continuous outcomes will be performed adjusted for covariates listed above using mixed-effects generalised linear models assuming a normal distribution and identity function, reported as mean difference (MD) with 95% confidence intervals.

13. Exploratory analyses

Enriched Sample Analysis

Where appropriate, data from outcomes obtained at the 6–8-year follow-up will be added in the outcomes if they are missing at 20-year and the denominators corrected to include the number of participants in each treatment arm for whom data were available at either 6–8-year or 20-year follow up. The feasibility of using the 6–8-year data as a prior in a Bayesian analysis will also be investigated.

Subgroup analyses

Exploratory analyses for primary and secondary outcomes will investigate if the treatment effect of antenatal corticosteroid is influenced by the following variables:

- Sex
- Socioeconomic status (based on New Zealand Deprivation Index for their area of residence at 20-year follow-up) – most deprived (deciles 8-10) vs other.

Analyses for each of the primary and secondary outcomes listed will be performed with each of these potential subgroups as main and interaction effects (with treatment allocation) and measures of effect will be presented for each of the subgroup strata.

Sensitivity analyses

The following sensitivity analyses will be performed:

- Excluding participants with asthma diagnosed <5 and no current asthma (subgroup of primary outcome).
- Excluding participants with respiratory outcomes derived from hospital admission data where the respiratory diagnosis was not the primary reason for admission (subgroup of composite outcome).

Post hoc analyses

Any post-hoc, exploratory analyses which were not identified in this SAP but are completed to support the listed analyses will be identified clearly.

14. References

1. Crowther CA, Haslam RR, Hiller JE, Doyle LW, Robinson JS, Australasian Collaborative Trial of Repeat Doses of Steroids Study Group. Neonatal respiratory distress syndrome after repeat exposure to antenatal corticosteroids: a randomised controlled trial. Lancet. 2006;367(9526):1913-9.

2. Crowther CA, Doyle LW, Haslam RR, Hiller JE, Harding JE, Robinson JS. Outcomes at 2 years of age after repeat doses of antenatal corticosteroids. New England Journal of Medicine. 2007;357(12):1179-89.
3. Crowther CA, Anderson PJ, McKinlay CJ, Harding JE, Ashwood PJ, Haslam RR, et al. Mid-childhood outcomes of repeat antenatal corticosteroids: a randomized controlled trial. Pediatrics. 2016;138(4):e20160947.
4. Cartwright RD, Crowther CA, Anderson PJ, Harding JE, Doyle LW, McKinlay CJD. Association of Fetal Growth Restriction With Neurocognitive Function After Repeated Antenatal Betamethasone Treatment vs Placebo: Secondary Analysis of the ACTORDS Randomized Clinical Trial. JAMA Netw Open. 2019;2(2):e187636.
5. McKinlay CJ, Cutfield WS, Battin MR, Dalziel SR, Crowther CA, Harding JE. Mid-childhood bone mass after exposure to repeat doses of antenatal glucocorticoids: a randomized trial. Pediatrics. 2017;139(5):e20164250.
6. McKinlay CJ, Cutfield WS, Battin MR, Dalziel SR, Crowther CA, Harding JE, et al. Cardiovascular risk factors in children after repeat doses of antenatal glucocorticoids: an RCT. Pediatrics. 2015;135(2):e405-15.
7. Shahbaz M, Gamble G, Harding JE. Comparison of outcomes of the 50-year follow-up of a randomized trial assessed by study questionnaire and by data linkage: The CONCUR Study. Clinical Trials (in press). 2024.
8. R Core Team. R: A Language and Environment for Statistical Computing. Vienna, Austria: R Foundation for Statistical Computing; 2023.

15. Appendix

15.1. Flow diagram

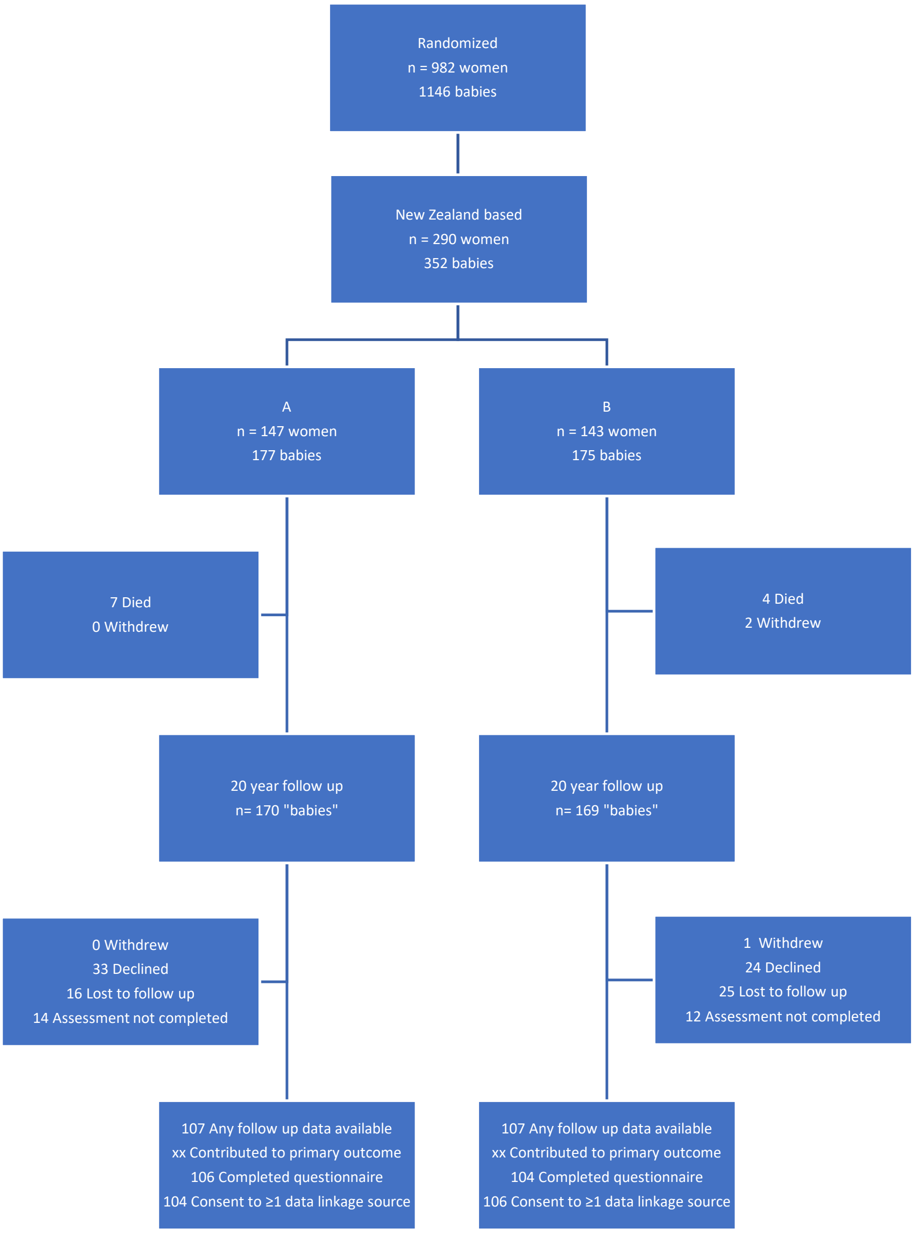
Randomized
n = 982 women
1146 babies
New Zealand based
n = 290 women
352 babies
A
n = 147 women
177 babies
B
n = 143 women
175 babies
7 Died
0 Withdrew
4 Died
2 Withdrew
20 year follow up
n= 170 "babies"
20 year follow up
n= 169 "babies"
0 Withdrew
33 Declined
16 Lost to follow up
14 Assessment not completed
1 Withdrew
24 Declined
25 Lost to follow up
12 Assessment not completed
107 Any follow up data available
xx Contributed to primary outcome
106 Completed questionnaire
104 Consent to ≥1 data linkage source
107 Any follow up data available
xx Contributed to primary outcome
104 Completed questionnaire
106 Consent to ≥1 data linkage source

15.2. Outcomes list

Primary Outcome

- Any asthma diagnosis

Secondary Outcomes:

- Asthma currently on treatment
- Death (any cause after randomisation)
- Respiratory outcomes (composite) – any respiratory diagnoses other than asthma including chronic lung disease
- Neurodevelopmental disability (composite) – any of:
  - visual impairment
  - hearing impairment
  - intellectual impairment
  - cerebral palsy
  - epilepsy
  - autism spectrum disorder (including Asperger's syndrome)
  - attention deficit disorder (ADD) or attention deficit hyperactivity disorder (ADHD)
- Cardiovascular outcomes (composite) – any cardiovascular diagnosis including hypertension, cardiomyopathies, arrhythmias, heart failure
- Number of cardiovascular disease risk factors (composite categorised as 0, 1 or >1 risk factors) – any of:
  - high blood pressure
  - dyslipidaemia
  - diabetes mellitus
  - obesity/overweight
- Diabetes outcomes (composite) – any of prediabetes, diabetes mellitus, gestational diabetes mellitus
- Mental health outcomes (composite) – any mental health diagnosis including depression, bipolar affective disorder, schizophrenia, anxiety disorders, suicide/self-harm
- Bone health outcomes: any diagnoses related to bone disease
- Bone health outcomes: number of fractures
- General health outcomes: self-reported general health (reported health fair or poor)
- General health outcomes: functional difficulties (categorised as no disability / moderate or non-severe disabilities / severe disabilities)
- General health outcomes: oral health (reported oral health fair or poor or tooth extractions)

Tertiary Outcomes:

- Educational achievement outcomes: no secondary school qualification
- Educational achievement outcomes: any disciplinary action
- Social outcomes: any convictions
- Social outcomes: unemployment
- Social outcomes: alcohol use in the past year
- Social outcomes: recreational drug use
- Social outcomes: past/current smoking (pack years)
- Social outcomes: past/current vaping
- Components of respiratory composite outcome:
  - Respiratory infections
  - Chronic respiratory conditions (excluding asthma)
  - Respiratory failure
  - Respiratory trauma
- Components of neurodevelopmental disability composite outcome:
  - visual impairment
  - hearing impairment
  - intellectual impairment
  - cerebral palsy
  - epilepsy
  - autism spectrum disorder (including Asperger's syndrome)

- attention deficit disorder (ADD) or attention deficit hyperactivity disorder (ADHD)
- Components of cardiovascular composite outcome:
  - Hypertension
  - Cardiomyopathies
  - Arrhythmias
  - heart failure
- Components of cardiovascular disease risk factors composite outcome:
  - high blood pressure
  - dyslipidaemia
  - diabetes mellitus
  - obesity/overweight
- Components of diabetes composite outcome:
  - prediabetes/diabetes mellitus
  - gestational diabetes mellitus
- Components of mental health composite outcome
  - depression
  - bipolar affective disorder
  - anxiety disorders
  - suicide/self-harm
  - schizophrenia
- Components of functional difficulties outcome
  - Difficulty seeing, even if wearing glasses
  - Difficulty hearing, even if using a hearing aid
  - Difficulty walking or climbing steps
  - Difficulty remembering or concentrating
  - Difficulty washing all over or dressing
  - Difficulty communicating
- Components of obesity/overweight outcome
  - BMI
  - height
  - weight

Study Outcome (definitions)	Priority Order for use of Data Sources	Details of data*:	Criteria for adjudication (if applicable)	Comments/ Reference:
Primary outcome				
**Any asthma diagnosis**<br>Defined as the presence of either of:<br>1. Self-report of a doctor's diagnosis of asthma.<br>2. Hospital admissions with diagnostic codes for asthma at any age.<br>3. Dispensing of pharmaceuticals for asthma at any age at least one repeat prescription more than 1 day apart.	Self-report questionnaire	B22. Have you ever been told by a doctor that you have asthma?	If answers no to question about asthma but has evidence from only one other data source, then will be adjudicated.	
	NMDS admissions data	Admissions with the following ICD-10AM codes:<br>J45.0 – J45.998 Asthma and subcodes.<br>J46 status asthmaticus<br>R06.2 Wheezing<br>U833 Asthma, without mention of chronic obstructive pulmonary disease		https://www.hqsc.govt.nz/assets/Our-data/Publications-resources/Methodology-asthma-update-2020.pdf
	NZ pharmaceuticals collection	Prescriptions + dates<br>**Short acting beta agonist (SABA):** Salbutamol (*Ventolin, respigen, SalAir)*, terbutaline (*Bricanyl*).<br>**Long acting beta agonists (LABA)**: Eformoterol fumarate (*Oxis, Foradil)*), salmeterol (*Serevent, Meterol),*<br>**Inhaled corticosteroid (ICS):** Beclometasone dipropionate (*Beclazone, Qvar*), budesonide		https://www.hqsc.govt.nz/assets/Our-data/Publications-resources/Methodology-asthma-update-2020.pdf

Study Outcome (definitions)	Priority Order for use of Data Sources	Details of data*:	Criteria for adjudication (if applicable)	Comments/ Reference:
		(*Pulmicort*), fluticasone propionate (*Flixotide, Floair*)<br>**Combination of LABA and ICS:** fluticasone + salmeterol (*Seretide, RexAir*), budesonide + formoterol (*Symbicort, DueResp Spiromax, Vannair),* fluticasone furoate + vilanterol *(Breo Ellipta, Relvar Ellipta)*<br>**Combination of SABA and SAMA:** Salbutamol + ipratropium bromide<br>**Oral Treatments**<br>Montelukast (*Montelukast Mylan, Apo-Montelukast, Montelukast Accord*)		
Secondary outcomes				
**Asthma currently on treatment**<br>Defined as the presence of either of:<br>1. Self-report of a doctor's diagnosis of asthma AND currently on treatment.<br>2. Hospital admissions with diagnostic codes for asthma at any age.<br>3. Dispensing of pharmaceuticals for asthma for at least one repeat prescription more	Self-report questionnaire	B22. Have you ever been told by a doctor that you have asthma?<br>B23. What treatments do you now have for asthma?	If answers no to question about asthma but has evidence from one other data source then will be adjudicated.	Timeframe of last 2 years to account for those with seasonal asthma who may only have received their last script last season.<br><br>Administrative data will be right censored at the date of the questionnaire for participants who completed the questionnaire and at the date of consent for those who did not.

Study Outcome (definitions)	Priority Order for use of Data Sources	Details of data*:	Criteria for adjudication (if applicable)	Comments/ Reference:
than 1 day apart and within last 2 years.				
	NMDS admissions data	Admissions with the following ICD-10AM codes:<br>J45.0 – J45.998 Asthma and subcodes.<br>J46 status asthmaticus<br>R06.2 Wheezing		https://www.hqsc.govt.nz/assets/Our-data/Publications-resources/Methodology-asthma-update-2020.pdf
	NZ pharmaceuticals collection	Prescriptions + dates<br>**Short acting beta agonist (SABA):** Salbutamol (*Ventolin, respigen, SalAir)*, terbutaline (*Bricanyl*).<br>**Long acting beta agonists (LABA)**: Eformoterol fumarate (*Oxis, Foradil)*), salmeterol (*Serevent, Meterol),*<br>**Inhaled corticosteroid (ICS):** Beclometasone dipropionate (*Beclazone, Qvar*), budesonide (*Pulmicort*), fluticasone propionate (*Flixotide, Floair*)<br>**Combination of LABA and ICS:** fluticasone + salmeterol (*Seretide, RexAir*), budesonide + formoterol (*Symbicort, DueResp Spiromax, Vannair),* fluticasone furoate + vilanterol *(Breo Ellipta, Relvar Ellipta)*<br>**Combination of SABA and SAMA:** Salbutamol + ipratropium bromide		https://www.hqsc.govt.nz/assets/Our-data/Publications-resources/Methodology-asthma-update-2020.pdf

Study Outcome (definitions)	Priority Order for use of Data Sources	Details of data*:	Criteria for adjudication (if applicable)	Comments/ Reference:
		**Oral Treatments**<br>Montelukast (*Montelukast Mylan, Apo-Montelukast, Montelukast Accord*)		
**Death (any cause after randomisation)**	MORT data	Death from any cause obtained from MORT dataset		
	Trial follow up records	Death from any cause as recorded in databases for original trial data and earlier follow-up studies.		
**Respiratory outcomes (composite)**<br>Defined as the presence of either of:<br>1. Self-report of a respiratory condition.<br>2. Hospital admissions with diagnostic codes for a respiratory condition at any age.	Self-report questionnaire	Free text response indicating a respiratory condition to one of the following questions:<br>B41. Do you currently have any other major illnesses?<br>B42. Have you ever had any other major illnesses in the past?		
	NMDS admissions data	Admissions with the following ICD-10AM codes:<br>J121 Respiratory syncytial virus pneumonia<br>J129 Viral pneumonia, unspecified<br>J13 Pneumonia due to Streptococcus pneumoniae<br>J150 Pneumonia due to Klebsiella pneumoniae		

Study Outcome (definitions)	Priority Order for use of Data Sources	Details of data*:	Criteria for adjudication (if applicable)	Comments/ Reference:
		J152 Pneumonia due to staphylococcus<br>J157 Pneumonia due to Mycoplasma pneumoniae<br>J159 Bacterial pneumonia, unspecified<br>J180 Bronchopneumonia, unspecified<br>J189 Pneumonia, unspecified<br>J201 Acute bronchitis due to Haemophilus influenzae<br>J208 Acute bronchitis due to other specified organisms<br>J210 Acute bronchiolitis due to respiratory syncytial virus<br>J218 Acute bronchiolitis due to other specified organisms<br>J219 Acute bronchiolitis, unspecified<br>J22 Unspecified acute lower respiratory infection<br>J47 Bronchiectasis<br>J690 Pneumonitis due to food and vomit<br>J869 Pyothorax without fistula<br>J90 Pleural effusion, not elsewhere classified<br>J91 Pleural effusion in conditions classified elsewhere<br>J939 Pneumothorax, unspecified		

Study Outcome (definitions)	Priority Order for use of Data Sources	Details of data*:	Criteria for adjudication (if applicable)	Comments/ Reference:
		J9565 Accidental puncture and laceration of pleura and diaphragm during a procedure<br>J958 Other postprocedural respiratory disorders<br>J9589 Other postprocedural complications and disorders of respiratory system, not elsewhere classified<br>J961 Chronic respiratory failure<br>J9619 Chronic respiratory failure, type unspecified<br>J969 Respiratory failure, unspecified<br>J981 Pulmonary collapse<br>J982 Interstitial emphysema<br>J984 Other disorders of lung<br>J985 Diseases of mediastinum, not elsewhere classified<br>J988 Other specified respiratory disorders<br>J998 Respiratory disorders in other diseases classified elsewhere<br>B371 Pulmonary candidiasis<br>B441 Other pulmonary aspergillosis<br>B460 Pulmonary mucormycosis<br>E840 Cystic fibrosis with pulmonary manifestations		

Study Outcome (definitions)	Priority Order for use of Data Sources	Details of data*:	Criteria for adjudication (if applicable)	Comments/ Reference:
		P271 Bronchopulmonary dysplasia originating in the perinatal period<br>P278 Other chronic respiratory diseases originating in the perinatal period<br>P279 Unspecified chronic respiratory disease originating in the perinatal period<br>Q336 Hypoplasia and dysplasia of lung<br>S270 Traumatic pneumothorax<br>S2731 Contusion and haematoma of lung<br>S2732 Laceration of lung		
**Neurodevelopmental disability (composite)**<br>Defined as the presence of neurodevelopmental disability either of:<br>1. Self-report of a neurodevelopmental disability.<br>2. Severe disability as classified by the Washington short set questions based on self-report questionnaire<br>3. Hospital admissions with diagnostic	Self-report questionnaire	Responded "A lot of difficulty" or "Can't do this at all" to any Washington Short Set questions:<br>F6. Do you have difficulty seeing, even if wearing glasses?<br>F7. Do you have difficulty hearing, even if using a hearing aid?<br>F8. Do you have difficulty walking or climbing steps?<br>F9. Do you have difficulty remembering or concentrating?<br>F10. Do you have difficulty washing all over or dressing?<br>F11. Using your usual language, do you have difficulty communicating, for example, understanding or being understood?		First, we recommend a three-way disaggregation of a) persons with no difficulty in all domains; b) persons with some difficulty in one or more domains; and c) persons with a lot of difficulty in one or more domains. The three groups are mutually exclusive. They could be labelled as persons without disability, persons with moderate or non-severe disabilities, and persons with severe disabilities respectively.<br>https://doi.org/10.1016/j.dhjo.2023.101499

Study Outcome (definitions)	Priority Order for use of Data Sources	Details of data*:	Criteria for adjudication (if applicable)	Comments/ Reference:
codes for a neurodevelopmental disability at any age.<br>4. Whaikaha claims relating to a neurodevelopmental disability at any age<br>5. Dispensing of pharmaceuticals for ADHD or epilepsy for at least one repeat prescription more than 1 day apart and within last 2 years .		Free text response indicating a neurodevelopmental disability to one of the following questions:<br>B41. Do you currently have any other major illnesses?<br>B42. Have you ever had any other major illnesses in the past?<br><br>D1. Have you ever been told by a doctor that you have autism spectrum disorder, including Asperger's syndrome?<br><br>D3. Have you ever been told by a doctor that you have attention deficit disorder (ADD) or attention deficit hyperactivity disorder (ADHD disorder)?<br><br>Free text response in tracking database indicating a neurodevelopmental disability severe enough that the questionnaire was completed by someone other than the participant.		
	NMDS admissions data	Admissions with the following ICD-10AM codes:<br>F840 Childhood autism<br>F900 Disturbance of activity and attention		

Study Outcome (definitions)	Priority Order for use of Data Sources	Details of data*:	Criteria for adjudication (if applicable)	Comments/ Reference:
		G8000 Spastic cerebral palsy, unspecified<br>G8001 Spastic diplegic cerebral palsy<br>G8002 Spastic hemiplegic cerebral palsy<br>G8003 Spastic quadriplegic cerebral palsy<br>G8009 Other spastic cerebral palsy<br>G808 Other infantile cerebral palsy<br>G809 Infantile cerebral palsy, unspecified<br>F719 Moderate mental retardation without mention of impairment of behaviour<br>F728 Severe mental retardation, other impairments of behaviour<br>F799 Unspecified mental retardation without mention of impairment of behaviour<br>F809 Developmental disorder of speech and language, unspecified<br>F89 Unspecified disorder of psychological development<br>H905 Sensorineural hearing loss, unspecified<br>U804 Cerebral palsy<br>U794 Disorder of intellectual development		

Study Outcome (definitions)	Priority Order for use of Data Sources	Details of data*:	Criteria for adjudication (if applicable)	Comments/ Reference:
		U803 Epilepsy		
	Whaikaha	Any neurodevelopmental disability listed as a principal disability		
	NZ pharmaceuticals collection	Prescriptions + dates<br>**ADHD stimulants:** Atomoxetine, Dexamfetamine sulfate, Methylphenidate hydrochloride, Methylphenidate hydrochloride extended-release<br>**Epilepsy:** Carbamazepine, Clobazam, Clonazepam, Gabapentin, Lacosamide, Lamotrigine, Levetiracetam, Phenytoin sodium, Pregabalin, Sodium valproate, Topiramate		ADHD pharms: https://nzfchildren.org.nz/nzfc_2328
**Cardiovascular outcomes (composite)**<br>Defined as the presence of either of:<br>1. Self-report of a cardiovascular condition.<br>2. Hospital admissions with diagnostic codes for a cardiovascular condition at any age.<br>3. Dispensing of a listed pharmaceutical for	Self-report questionnaire	B1. Have you ever been told by a doctor that you have high blood pressure?<br>B5. Have you ever been told by a doctor that you have had a heart attack?<br>B12. Have you ever been told by a doctor that you have heart failure?<br>B13. Have you ever been told by a doctor that you have any other heart condition/s?<br><br>Free text response indicating a cardiovascular condition to one of the following questions:	May need to look at all of these as an adjudication committee	

Study Outcome (definitions)	Priority Order for use of Data Sources	Details of data*:	Criteria for adjudication (if applicable)	Comments/ Reference:
treatment of hypertension		B41. Do you currently have any other major illnesses?<br>B42. Have you ever had any other major illnesses in the past?		
	NMDS admissions data	Admissions with the following ICD-10AM codes:<br>I151 Hypertension secondary to other renal disorders<br>I158 Other secondary hypertension<br>I420 Dilated cardiomyopathy<br>I451 Other and unspecified right bundle-branch block<br>I471 Supraventricular tachycardia<br>I490 Ventricular fibrillation and flutter<br>I495 Sick sinus syndrome<br>I499 Cardiac arrhythmia, unspecified<br>I500 Congestive heart failure<br>I517 Cardiomegaly<br>I518 Other ill-defined heart diseases<br>I822 Embolism and thrombosis of vena cava<br>I828 Embolism and thrombosis of other specified veins<br>I978 Other postprocedural disorders of circulatory system, not elsewhere classified		

Study Outcome (definitions)	Priority Order for use of Data Sources	Details of data*:	Criteria for adjudication (if applicable)	Comments/ Reference:
		I9789 Other postprocedural complications and disorders of the circulatory system, not elsewhere classified<br>U822 Chronic heart failure		
	NZ pharmaceuticals collection	**High blood pressure**<br>*Angiotensin converting enzyme (ACE) inhibitors*<br>(i.e. Captopril, Cilazapril, Enalapril, Lisinopril, Perindopril, quinapril)<br>*Angiotensin receptor blockers (ARBs)* (i.e. Candesarten, Losarten, Irbesarten)<br>*Beta blockers* (Atenolol, bisoprolol, carvedilol, celiprolol, labetalol, metoprolol, propranolol, sotalol)<br>*Diuretics* (hydrochlorothiazide, bendroflumathiazide, chlorthalidone, indapamide)<br>*Calcium channel blockers* (amlodipine, diltiazem, felodipine, nifedipine)<br>*Combination* (inhibace plus, accuretic, Arrow-Losartan & Hydrochlorothiazide)		
**Number of cardiovascular disease risk factors**	Self-report questionnaire	B1. Have you ever been told by a doctor that you have high blood pressure?		

Study Outcome (definitions)	Priority Order for use of Data Sources	Details of data*:	Criteria for adjudication (if applicable)	Comments/ Reference:
**(composite categorised as 0, 1 or >1 risk factors) – any of:**<br>• **high blood pressure**<br>• **dyslipidaemia**<br>• **diabetes mellitus** (see following outcome)<br>• **obesity/overweight**<br><br>Defined as the presence of either of:<br>1. Self-report of a risk factor listed above.<br>2. Hospital admissions with diagnostic codes for a risk factor listed above.<br>3. Testsafe lab results of dyslipidaemia<br>4. Dispensing of a listed pharmaceutical for treatment of dyslipidaemia or hypertension		B3. Have you ever been told by a doctor that you have high blood cholesterol levels?<br>B18. Have you ever been told by a doctor that you have pre-diabetes or diabetes? (not including gestational diabetes)<br><br>BMI >25 as calculated using:<br>What is your current weight? (Q.F2)<br>What is your current height (Q.F3)		
	NMDS admissions data	Admissions with the following ICD-10AM codes:<br>**Hypertension:**<br>Essential hypertension; I110, I119 hypertensive heart disease; I120,		

Study Outcome (definitions)	Priority Order for use of Data Sources	Details of data*:	Criteria for adjudication (if applicable)	Comments/ Reference:
		I129 Hypertensive renal disease; I13x Hypertensive heart and renal disease, I15x secondary hypertension<br><br>**Diabetes**<br>E10 (Type 1 diabetes mellitus), E11 (Type 2 diabetes mellitus), E13 (Other diabetes mellitus), E14 (diabetes mellitus unspecified), O24.0 (pre-existing Type 1 diabetes mellitus during pregnancy), O24.1 (pre-existing type 2 diabetes mellitus), O24.2 (pre-existing malnutrition associated diabetes mellitus in pregnancy), O24.3 (pre-existing diabetes mellitus unspecified in pregnancy).<br><br>**Obesity**<br>U781 Obesity		
	Testsafe lab results	Lipid profile tests and dates		Dyslipidaemia, defined as TC >5 mmol/L, LDL ≥3.4 mmol/L, Triglycerides ≥2 mmol/L or HDL <1.0 mmol/L.<br><br>Stone NJ, Robinson JG, Lichtenstein AH, Bairey Merz CN, Blum CB, Eckel RH, et al. 2013 ACC/AHA Guideline on the Treatment of Blood Cholesterol to Reduce Atherosclerotic Cardiovascular Risk in Adults: A Report of the American College of Cardiology/American Heart Association Task Force on Practice Guidelines. Circulation [Internet]. 2014 Jun 24 [cited 2023 Jun 15];129(25_suppl_2). Available from: https://doi.org/10.1161/01.cir.0000437738.63853.7a

Study Outcome (definitions)	Priority Order for use of Data Sources	Details of data*:	Criteria for adjudication (if applicable)	Comments/ Reference:
	NZ pharmaceuticals collection	**High blood pressure**<br>*Angiotensin converting enzyme (ACE) inhibitors*<br>(i.e. Captopril, Cilazapril, Enalapril, Lisinopril, Perindopril, quinapril)<br>*Angiotensin receptor blockers (ARBs)* (i.e. Candesarten, Losarten, Irbesarten)<br>*Beta blockers* (Atenolol, bisoprolol, carvedilol, celiprolol, labetalol, metoprolol, propranolol, sotalol)<br>*Diuretics* (hydrochlorothiazide, bendroflumathiazide, chlorthalidone, indapamide)<br>*Calcium channel blockers* (amlodipine, diltiazem, felodipine, nifedipine)<br>*Combination* (inhibace plus, accuretic, Arrow-Losartan & Hydrochlorothiazide)<br><br>**Dyslipidaemia**<br>*Statins* (Atorvastatin, Pravastatin, Rosuvastatin, Simvastatin)<br>*Fibrate* (bezafibrate)<br>*Niacin derivatives* (acipimox)<br>Ezetimibe		

**Study Outcome (definitions)**	**Priority Order for use of Data Sources**	**Details of data*:**	**Criteria for adjudication (if applicable)**	**Comments/ Reference:**
		Colestipol		
**Diabetes outcomes (composite)**<br>Defined as the presence of either of:<br>1. Self-report of diabetes or gestational diabetes.<br>2. Hospital admission with a diagnosis of diabetes mellitus<br>3. Prescription of pharmaceuticals for treatment of diabetes mellitus<br>**4.** Testsafe Laboratory results diagnostic of diabetes mellitus	Self-report questionnaire	B18. Have you ever been told by a doctor that you have pre-diabetes or diabetes? (not including gestational diabetes)<br>C9. During your pregnancy/ies did a doctor ever tell you that you had developed gestational diabetes?	If answers "No" to question about diabetes mellitus but has 2 or more other data sources suggesting diabetes mellitus, then classify as DM. If answers no to question about DM but has only one piece of information, then case will be adjudicated using available information.<br>If answers "yes" but no diabetes treatment (self-report or NZ pharmaceuticals collection), will be adjudicated.	
	NMDS admissions data	Hospital admissions with a diagnosis of diabetes mellitus. This includes the following ICD 10 codes:		

Study Outcome (definitions)	Priority Order for use of Data Sources	Details of data*:	Criteria for adjudication (if applicable)	Comments/ Reference:
		E10 (Type 1 diabetes mellitus), E11 (Type 2 diabetes mellitus), E13 (Other diabetes mellitus), E14 (diabetes mellitus unspecified), O24.0 (pre-existing Type 1 diabetes mellitus during pregnancy), O24.1 (pre-existing type 2 diabetes mellitus), O24.2 (pre-existing malnutrition associated diabetes mellitus in pregnancy), O24.3 (pre-existing diabetes mellitus unspecified in pregnancy), O24.4 (Diabetes mellitus arising during pregnancy)..		
	NZ pharmaceuticals collection	Prescription for any of the following*<br>Metformin (ie. Galvumet or Galvus (vildagliptin))<br>*Insulin* (rapid-acting: NovoRapid, Apidra, Apidra Solostar, Humalog)<br>(short-acting: Actrapid, Humulin R)<br>(intermediate and long acting: isophane/Protophane, Humulin NPH, Lantus, Lantus Solostar, detemeir/Levemir)	If dispensed metformin and is male, then classify as diabetes mellitus. If dispensed metformin, is female and answers “no” to polycystic ovarian syndrome (PCOS) in questionnaire then classify as diabetes	

Study Outcome (definitions)	Priority Order for use of Data Sources	Details of data*:	Criteria for adjudication (if applicable)	Comments/ Reference:
			mellitus. If "yes" or did not answer PCOS question, then will be adjudicated.	
	Testsafe lab results	HbA1c results + dates<br>Glucose tests + dates<br>GTT results and tests		**New Zealand Society for the Study of Diabetes 2021 definition of diabetes mellitus.**[1]<br>If symptomatic diagnosed by<br>1.HbA1c ≥ 50 mmol/mol, or<br>2. Fasting glucose ≥ 7 mmol/L or<br>3. Random glucose ≥ 11.1 mmol/L.<br><br>If asymptomatic diagnosed by<br>1. Two abnormal tests [HbA1c (≥ 50 mmol/mol), or fasting glucose ≥ 7 mmol/L, or random glucose ≥ 11.1 mmol/L] either on same day or subsequent test without delay.<br><br>2. If OGTT<br>Fasting glucose ≥ 7 mmol/L or 2 hour glucose is ≥ 11.1 mmol/L.
**Mental health outcomes (composite)**<br>Defined as the presence of either of:<br>1. Self-report of a mental health diagnosis.<br>2. Hospital admission with a mental health diagnosis	Self-report questionnaire	D5. Have you ever been told by a doctor that you have depression?<br>D7. Have you ever been told by a doctor that you have bipolar disorder, which is sometimes called manic depression?<br>D9. Have you ever been told by a doctor that you have anxiety disorder? This includes panic attacks, phobia, post-traumatic		

Study Outcome (definitions)	Priority Order for use of Data Sources	Details of data*:	Criteria for adjudication (if applicable)	Comments/ Reference:
3. Prescription of pharmaceuticals for treatment of mental health diagnosis<br>4. ACC data relating to suicide/self-harm		stress disorder, and obsessive/compulsive disorder.<br><br>Free text response indicating a mental health diagnosis to one of the following questions:<br>B41. Do you currently have any other major illnesses?<br>B42. Have you ever had any other major illnesses in the past?		
	NMDS admissions data	F320-F329: Depressive episode<br>F330-F339: Recurrent depressive disorder<br>F310-F319: Bipolar affective disorder<br>F400-F409: Phobic anxiety disorders<br>F410-F419: Other anxiety disorders<br>F10-F19: Mental and behavioural disorders due to psychoactive substance use.<br>F20-F29: Schizophrenia, schizotypal, delusional, and other non-mood psychotic disorders.<br>F30-F39: Mood (affective) disorders.<br>F40-F48: Anxiety, dissociative, stress-related, somatoform and other nonpsychotic mental disorders.<br>F50: eating disorders.		

Study Outcome (definitions)	Priority Order for use of Data Sources	Details of data*:	Criteria for adjudication (if applicable)	Comments/ Reference:
		F60-F69: Disorders of adult personality and behaviour.<br>F80-F89: Pervasive and specific developmental disorders<br>F90-F98: Behavioural and emotional disorders with onset usually occurring in childhood and adolescence.<br>F99: unspecified mental disorder.<br>U793 Depression		
	NZ pharmaceuticals collection	**MHA medications List:**<br>1011 Risperidone, 1030 Sertraline hydrochloride, 1069 Amoxapine, 1078 Clozapine, 1080 Amylobarbitone sodium, 1125 Nefazodone, 1140 Olanzapine, 1166 Bromazepam,<br>1180 Venlafaxine, 1183 Quetiapine, 1183 Quetiapine fumarate, 1193 Citalopram hydrobromide, 1315 Clomipramine hydrochloride,<br>1432 Disulfiram, 1437 Dothiepin hydrochloride, 1438 Doxepin hydrochloride,<br>1532 Flupenthixol decanoate<br>1533 Fluphenazine decanoate<br>1535 Fluphenazine hydrochloride<br>1642 Imipramine hydrochloride<br>1729 Loprazolam mesylate<br>1731 Lormetazepam<br>1732 Loxapine succinate<br>1760 Maprotiline hydrochloride		https://www-ncbi-nlm-nih-gov.ezproxy.auckland.ac.nz/pmc/articles/PMC7045433/<br>Bowden, N., Gibb, S., Thabrew, H. et al. Case identification of mental health and related problems in children and young people using the New Zealand Integrated Data Infrastructure. BMC Med Inform Decis Mak 20, 42 (2020).<br>- reference for a case identification method of using the IDI for mental health research.

Study Outcome (definitions)	Priority Order for use of Data Sources	Details of data*:	Criteria for adjudication (if applicable)	Comments/ Reference:
		1780 Meprobamate<br>1824 Mianserin hydrochloride<br>1911 Oxazepam<br>1950 Pericyazine<br>1955 Phenelzine sulphate<br>1990 Pimozide<br>1994 Pipothiazine palmitate<br>2255 Thioridazine hydrochloride<br>2260 Thiothixene<br>2285 Tranylcypromine sulphate<br>2295 Triazolam<br>2301 Trimipramine maleate<br>2466 Lithium carbonate<br>2632 Alprazolam<br>2636 Fluoxetine hydrochloride<br>2638 Moclobemide<br>2820 Fluspirilene<br>3753 Mirtazapine<br>3785 Venlafaxine<br>3793 Naltrexone hydrochloride<br>3873 Ziprasidone<br>3878 Aripiprazole<br>3884 Amisulpride<br>3923 Donepezil hydrochloride<br>3926 Escitalopram<br>3927 Sertraline<br>6006 Buspirone hydrochloride<br>6009 Paroxetine hydrochloride		
	ACC	ACC read codes<br>TK xx Suicide and self-inflicted injury		

**Study Outcome (definitions)**	**Priority Order for use of Data Sources**	**Details of data*:**	**Criteria for adjudication (if applicable)**	**Comments/ Reference:**
**Bone health outcomes: any diagnoses related to bone disease**<br>Defined as the presence of either of:<br>1. Self-report of bone disease.<br>2. Hospital admission with a diagnosis of bone disease	Self-report questionnaire	B32. Have you ever been told by a doctor that you have a bone or joint disease or condition?<br><br>B33. Have you ever experienced any of the following bone or joint conditions?<br><br>Free text response indicating bone disease to one of the following questions:<br>B41. Do you currently have any other major illnesses?<br>B42. Have you ever had any other major illnesses in the past?		
	NMDS admissions data	U864 Osteoporosis<br>M8088 Other osteoporosis with pathological fracture, other		
**Bone health outcomes: number of fractures**<br>Defined as the presence of either of:<br>1. Self-report of number of fractures<br>2. Number of hospital admission / ED treatments for a bone fracture<br>3. ACC data relating to fractures	Self-report questionnaire	B30. Have you ever had a bone fracture (broken bone)?<br>B30.1. How many fracture(s) can you recall?		

Study Outcome (definitions)	Priority Order for use of Data Sources	Details of data*:	Criteria for adjudication (if applicable)	Comments/ Reference:
	NMDS	**ICD-10AM codes:**<br>S020-S024, S026, S028-S029: Fracture of the skull and facial bones excluding fracture of tooth.<br>S120-S126, S128-S129: Fracture of cervical vertebra and other parts of neck<br>S220 Fracture of thoracic vertebra<br>S222: Fracture of sternum<br>S223: Fracture of one rib<br>S224: Fracture of multiple ribs<br>S225: flail chest<br>S229: Fracture of bony thorax, part unspecified<br>S32: Fracture of lumbar spine and pelvis<br>S42: Fracture of shoulder and upper arm<br>S52: Fracture of forearm<br>S62: Fracture at wrist and hand level<br>S72: Fracture of femur<br>S82: Fracture of lower leg, including ankle<br>S92: Fracture of foot and toe, except ankle		
	ACC	ACC read codes<br>S0 - S3z00 All fractures		

Study Outcome (definitions)	Priority Order for use of Data Sources	Details of data*:	Criteria for adjudication (if applicable)	Comments/ Reference:
**General health outcomes: self-reported general health** (reported health fair or poor)	Self-report questionnaire	E1. In general, would you say your health is ...		
**General health outcomes: functional difficulties** (categorised as no disability / moderate or non-severe disabilities / severe disabilities)	Self-report questionnaire	Washington Short Set questions:<br>F6. Do you have difficulty seeing, even if wearing glasses?<br>F7. Do you have difficulty hearing, even if using a hearing aid?<br>F8. Do you have difficulty walking or climbing steps?<br>F9. Do you have difficulty remembering or concentrating?<br>F10. Do you have difficulty washing all over or dressing?<br>F11. Using your usual language, do you have difficulty communicating, for example, understanding or being understood?		First, we recommend a three-way disaggregation of a) persons with no difficulty in all domains; b) persons with some difficulty in one or more domains; and c) persons with at least a lot of difficulty in one or more domains. The three groups are mutually exclusive. They could be labelled as persons without disability, persons with moderate or non-severe disabilities, and persons with severe disabilities respectively.<br>https://doi.org/10.1016/j.dhjo.2023.101499
**General health outcomes: oral health** (reported oral health fair or poor or tooth extractions)<br>Defined as the presence of either of:<br>1. Self-report of oral health that is fair or poor<br>2. Self-report of one or more teeth removed	Self-report questionnaire	F4. How would you describe the health of your teeth or mouth?<br>F5. Have you had any teeth removed for tooth decay or gum disease? (Do not include teeth lost for other reasons such as injury, crowded mouth or orthodontics.)<br>F5.1. How many of your teeth have been removed by a dental health care worker because of tooth decay, an abscess, infection or gum disease?		

Study Outcome (definitions)	Priority Order for use of Data Sources	Details of data*:	Criteria for adjudication (if applicable)	Comments/ Reference:
for tooth decay or gum disease				
Tertiary Outcomes				
**Educational achievement outcomes: no secondary school qualification**<br>Defined as the presence of either of:<br>1. Self-report of educational achievement.<br>2. NZQA record of highest qualification	Self-report questionnaire	G3. What is your highest secondary school qualification?		
	NZQA	No record of NCEA 2 or higher		
**Educational achievement outcomes: any disciplinary action**<br>Defined as the presence of MoE record of any stand-downs, suspensions, exclusions and expulsions from educational institutions	MoE	MoE record of any stand-downs, suspensions, exclusions and expulsions		
**Social outcomes: any convictions**	MOJ convictions data	Data on convictions		

Study Outcome (definitions)	Priority Order for use of Data Sources	Details of data*:	Criteria for adjudication (if applicable)	Comments/ Reference:
Defined as the presence of MoJ record of any convictions				
**Social outcomes: unemployment**	Self-report questionnaire	G5. Which of these statements best describes your current work situation?		
**Social outcomes: alcohol use** Defined as the presence of either of: 1. Self-report of drinking alcohol in the past year	Self-report questionnaire	H11. Have you had a drink containing alcohol in the last year?		New Zealand Health Survey Indicators (Prevalence or mean for 2022/23) Past-year drinkers (had alcoholic drink in the past 12 months) (76.3%) https://minhealthnz.shinyapps.io/nz-health-survey-2022-23-annual-data-explorer/_w_9cd942d6/#!/explore-topics
**Social outcomes: recreational drug use** Defined as the presence of either of: 1. Self-report of recreational drug use in the past year	Self-report questionnaire	H13. In the last 12 months, have you used any of the following drugs for recreational or non-medical purposes, or to get high?		
**Social outcomes: past/current smoking (pack years)**	Self-report questionnaire	H1. Do you currently smoke? H2. How many cigarettes do you smoke daily? H3. Have you ever been a smoker in the past? H4. How many cigarettes did you smoke daily in the past?		

**Study Outcome (definitions)**	**Priority Order for use of Data Sources**	**Details of data*:**	**Criteria for adjudication (if applicable)**	**Comments/ Reference:**
		H5. Do you know how many years in your life have you/did you smoke daily or almost daily? Specify the length (Please give number of years)		
**Social outcomes: past/current vaping**	Self-report questionnaire	H6. Do you currently vape?<br>H8. Have you ever been a vaper in the past?		Very hard to quantify vaping e.g. puffs per day see:<br>https://doi.org/10.1093/ntr/ntab074<br>https://doi.org/10.1136/tobaccocontrol-2021-056483

* Administrative data will be right censored at the date of the questionnaire for participants who completed the questionnaire and at the date of consent for those who did not.

Addendum to Statistical Analysis Plan ANCHOR ACTORDS v1.4 19 April 2024

Date: 29 May 2024

Addendum to statistical analysis plan for ACTORDS Follow up

Note: Versions 1.0-3 were early drafts before approval by the steering group. Version 1.4 was approved by the steering group and signed off on 14 May 2024.

The following text will be added to Appendix 15.2 Outcomes list:

- Dispensing of a listed pharmaceutical for treatment of hypertension as part of hypertension diagnosis as part of cardiovascular composite outcome
- Free text response in tracking database indicating a neurodevelopmental disability severe enough that the questionnaire was completed by someone other than the participant included in the definition of the neurodevelopmental composite outcome.

The following text was removed from Appendix 15.2 Outcomes list detailed definitions:

- Hypotension and associated NMDS codes were removed as a component of the cardiovascular composite outcome.
- Questionnaire item "B15. Have you ever been told by a doctor that you have had a stroke?" removed from definition of cardiovascular composite outcome to make this outcome consistent with cardiovascular composite outcomes in Section 5 and in the outcomes list at the start of Appendix 15.2

The following was corrected in Appendix 15.2 Outcomes list:

- For the definition of bipolar disorder as part of mental health composite outcome, questionnaire item "D8. What treatments do you now have for bipolar disorder?" corrected as "D7. Have you ever been told by a doctor that you have bipolar disorder, which is sometimes called manic depression?"

Addendum to Statistical Analysis Plan ANCHOR ACTORDS v1.5 29 May 2024

Date: 31 July 2024

Addendum to statistical analysis plan for ACTORDS Follow up

The following were added as tertiary outcomes:

- Components of functional difficulties outcome
    - Difficulty seeing, even if wearing glasses
    - Difficulty hearing, even if using a hearing aid
    - Difficulty walking or climbing steps
    - Difficulty remembering or concentrating
    - Difficulty washing all over or dressing
    - Difficulty communicating
- Components of obesity/overweight outcome
    - BMI
    - height
    - weight

This addition was made after all analyses had been completed but before unblinding of treatment groups.
